# Loss of *Parp7* increases type I interferon signalling and reduces pancreatic tumour growth by enhancing immune cell infiltration

**DOI:** 10.3389/fimmu.2024.1513595

**Published:** 2025-01-10

**Authors:** Vinicius Kannen, Marit Rasmussen, Siddhartha Das, Paolo Giuliana, Fauzia N. Izzati, Hani Choksi, Linnea A. M. Erlingsson, Ninni E. Olafsen, Samaneh S. Åhrling, Paola Cappello, Indrek Teino, Toivo Maimets, Kristaps Jaudzems, Antanas Gulbinas, Zilvinas Dambrauskas, Landon J. Edgar, Denis M. Grant, Jason Matthews

**Affiliations:** ^1^ Department of Pharmacology and Toxicology, University of Toronto, Toronto, ON, Canada; ^2^ Department of Nutrition, Institute of Basic Medical Sciences, Faculty of Medicine, University of Oslo, Oslo, Norway; ^3^ Department of Molecular Biotechnology and Health Sciences, University of Turin, Turin, Italy; ^4^ Institute of Molecular and Cell Biology, University of Tartu, Tartu, Estonia; ^5^ Latvian Institute of Organic Synthesis, Riga, Latvia; ^6^ Surgical Gastroenterology Laboratory, Institute for Digestive Research, Lithuanian University of Health of Sciences, Kaunas, Lithuania; ^7^ Department of Chemistry, University of Toronto, Toronto, ON, Canada; ^8^ Department of Immunology, University of Toronto, Toronto, ON, Canada

**Keywords:** Poly-ADP-ribose polymerase 7, type I interferon, pancreatic cancer, CRISPR/Cas9, tumour infiltrating leukocytes

## Abstract

**Background:**

Pancreatic ductal adenocarcinoma (PDAC) is one of the most lethal forms of cancer, and despite low incidence rates, it remains the sixth leading cause of cancer related deaths worldwide. Immunotherapy, which aims to enhance the immune system’s ability to recognize and eliminate cancer cells, has emerged as a promising approach in the battle against PDAC. PARP7, a mono-ADP-ribosyltransferase, is a negative regulator of the type I interferon (IFN-I) pathway and has been reported to reduce anti-tumour immunity.

**Methods:**

We used murine pancreatic cancer cells, CR705, CRISPR/Cas9, in vivo tumour models and spectral flow cytometry to determine the role of PARP7 in pancreatic tumour growth.

**Results:**

Loss of Parp7 elevated the levels of interferon stimulated gene factor 3 (ISGF3) and its downstream target genes, even in the absence of STING. Cancer cells knocked out for Parp7 (CR705Parp7KO) produced smaller tumours than control cells (CR705Cas9) when injected into immunocompetent mice. Transcriptomic analyses revealed that CR705Parp7KO tumours had increased expression of genes involved in immunoregulatory interactions and interferon signalling pathways. Characterization of tumour infiltrating leukocyte (TIL) populations showed that CR705Parp7KO tumours had higher proportions of natural killer cells, CD8+ T cells and a lower proportion of anti-inflammatory macrophages (M2). The overall TIL profile of CR705Parp7KO tumours was suggestive of a less suppressive microenvironment.

**Conclusions:**

Our data show that loss of Parp7 reduces PDAC tumour growth by increasing the infiltration of immune cells and enhancing anti-tumour immunity. These findings provide support to pursue PARP7 as a therapeutic target for cancer treatment.

## Introduction

Pancreatic ductal adenocarcinoma (PDAC), which represents more than 90% of pancreatic cancers (PC), is a highly aggressive disease with a poor prognosis (1). The disease arises from the uncontrolled growth of malignant cells in the pancreas, an essential organ responsible for producing digestive enzymes and hormones, including insulin. Despite low incidence rates, PDAC remains the sixth leading cause of cancer-related deaths worldwide (2). Due to its anatomical location, PDAC is characterized by its silent progression, often remaining undetected until advanced stages, resulting in poor prognoses and high mortality rates (3). Although there have been significant advancements in PDAC research, the mortality to incidence ratio has changed little over the past few decades. Compared with other cancers, PDAC exhibits remarkable resistance to conventional therapies and possesses a highly immunosuppressive tumour microenvironment (TME), enabling cancer cells to “hide” from the immune system (4). Surgery with curative intent together with adjuvant chemotherapy is the treatment of choice; however, this is only possible in about 10-20% of patients (5). Recent progress in targeting the immune system for cancer treatment, referred to as cancer immunotherapy, has caused a paradigm shift in therapeutic options for cancer patients. One of the most studied strategies involves targeting the immunosuppressive interaction between programmed death ligand 1 (PD-L1), which is present on tumour cells, and its receptor, programmed death receptor (PD-1), which is expressed on immune cells, such as activated T cells, natural killer (NK) cells, B cells, macrophages and different subsets of dendritic cells (DCs) (6). Inhibition of the PD-1/PD-L1 immune checkpoint axis has produced impressive response rates in various malignancies, such as melanoma, renal and lung cancer. However, despite PD-L1 being expressed in human PC samples, immunotherapy targeting PDAC has so far been ineffective due to reduced immune cell infiltration (7, 8). Thus, increased engagement of the immune response may provide significant clinical benefit for PDAC patients.

Type I interferons (IFN-Is) are cytokines that are released in response to pathogen or damage associated molecular patterns (PAMPs or DAMPs, respectively). They are expressed by almost all cells in the body and are involved in the regulation of many biological processes, such as cellular immune responses to infections, cell cycle regulation, differentiation and apoptosis (9). Nucleic acids released from damaged cells function as DAMPs and are recognized by pattern recognition receptors (PRRs), thereby eliciting an immune response. The presence of cytosolic DNA activates cyclic GMP-AMP synthase (cGAS), which synthesizes cyclic guanosine monophosphate-adenosine monophosphate (cGAMP). cGAMP subsequently activates stimulator of interferon response cGAMP interactor (STING) resulting in activation of TANK binding kinase 1 (TBK1), which phosphorylates IFN regulatory factor 3 (IRF3). IRF3 homodimerizes, translocates to the nucleus, and upregulates expression of IFN-Is, such as IFNβ, which is secreted from the cell (10). IFNβ binds to the IFNα/β receptor (IFNAR) complex on immune and non-immune cells, resulting in activation of signal transducer and activator of transcription 1 (STAT1) and STAT2. These proteins in turn form a complex with interferon regulatory factor 9 (IRF9) known as IFN stimulated gene factor 3 (ISGF3), which regulates the expression of IFN stimulated genes (ISGs) that play important roles in immunity (11). STING has emerged as a target for cancer therapy, providing new strategies to exploit the immune system to combat cancer (12). The expression levels of IFN-Is and other inflammatory cytokines downstream of STING activation contribute to enhanced anti-tumour immune responses (13). By utilizing this mechanism, STING agonists have been shown to induce tumour regression by enhancing the ability of immune cells to target cancer cells (14). IFN-Is regulate tumour infiltrating immune cells and are critically important in maintaining antigen-presenting lymphocyte function for effective anti-tumour immunity. They also exhibit cancer cell intrinsic properties, such as growth inhibition and increased apoptosis (11). Increasing IFN-I levels has shown promising results in the preclinical and clinical setting, and some studies report that they synergize with immune checkpoint inhibitors to reduce tumour growth in cancer models (15–18). In support of these findings, loss of IFN-I signalling enhances tumorigenesis and impairs anti-tumour responses (19). However, there is evidence that STING-mediated increases in IFN-I signalling induce pro-tumorigenic effects (20). STING activation in tumour cells has been reported to induce epithelial-mesenchymal transition (EMT) and promote tumour metastasis (21). IFN-Is induce PD-1 and PD-L1 levels in immune sand tumour cells. Radiation-induced IFN-I responses not only activate cytotoxic T cells but can also protect tumours from killing by cytotoxic T cells (22, 23).

PARP7, also known as 2,3,7,8-tetrachlorodibenzo-*p*-dioxin (TCDD)-inducible poly-ADP-ribose polymerase (TIPARP) is a member of the ADP-ribosyltransferase diphtheria-like (ARTD) family and a critical regulator of innate immune signalling (24). PARP7 uses NAD^+^ to transfer one molecule of ADP-ribose to specific amino acid residues on itself and on target proteins, in a process referred to as mono-ADP-ribosylation (MARylation) (25). MARylation is a reversible post-translational modification involved in several biological processes, such as immune cell function, transcriptional regulation, and DNA repair (26). PARP7 mRNA expression is regulated by several transcription factors, including the ligand-induced transcription factor aryl hydrocarbon receptor (AHR). In turn, PARP7 acts as a negative regulator of AHR signalling involving the MARylation of AHR (27–29). Previous studies have reported that PARP7 is required for AHR-dependent repression of IFN-I responses during viral infection associated with PAMP stimulation, a process that requires its catalytic activity (30). The repressive actions of PARP7 have been attributed to its ability to MARylate TBK1, thus preventing the downstream upregulation of IFN-Is (30). More recent studies provide evidence that PARP7 regulates IFN-I signalling downstream of TBK1 (31) and by targeting nuclear factor kappa B (32).

Treatment with IFN-I inducers in combination with immune checkpoint inhibitors has been shown to sustain anti-tumour responses in models of aggressive cancers (10). Recent studies in preclinical mouse models, showed that PARP7 inhibition (PARP7i) with RBN-2397 reduced CT26 colon tumour growth in immunocompetent mice, which was dependent on IFN-I signalling and that cotreatment of RBN-2397 with anti-PD1 further reduced tumour growth compared with either treatment alone (33). Consistent with these findings, we reported that injection of mice with murine EO771 breast cancer cells in which PARP7 was knocked out (Parp7^KO^) resulted in >80% reduced tumour growth in *Parp7* deficient mice compared with injected wildtype (WT) EO771 cells in WT mice (32). This was due to an increased infiltration of tumour-associated immune cells, resulting in augmented anti-tumour immunity. These findings show that PARP7 loss or its inhibition reduces tumour growth in different preclinical models by increasing anti-tumour responses. However, PARP7’s involvement in PDAC remains unclear.

In this study, we show that loss of PARP7 expression or its activity increases basal ISG expression levels in murine pancreatic cancer cells *in vitro*, and we demonstrate that *Parp7* loss decreases tumour growth through increased tumour infiltrating immune cells and enhanced anti-tumour immunity. Our results suggest that targeting PARP7 alone or combination with other immunotherapies should be considered as a new therapeutic strategy against PDAC.

## Materials and methods

### Chemicals and plasmids

DMSO was purchased from Sigma Aldrich (St. Louis, MO, USA), DMXAA from Invivogen (San Diego, CA, USA), RBN-2397 from MedChemExpress (Monmouth Junction, NJ, USA), 6-formylindolo(3,2-b)carbazole (FICZ) from SelleckChem (Houston, TX, USA), and IFNβ from R&D Systems (Minneapolis, MN, USA). The pSpCas9(BB)-2A-Puro (PX459) plasmid was purchased from Addgene (plasmid #62988) (Watertown, MA, USA).

### Cell culture

CR705 cells derived from a spontaneous pancreatic tumour in a *LSL-Kras^G12D/+^;LSL-Trp53^R172H/+^;Pdx1-Cre* (KPC) mouse were used in this study (34). K8484 cells were derived from KPC mice on the mixed 129/SvJae/C57BL/6 background. BxPC3 cells (CRL-1687) were purchased from ATCC. All cell lines were maintained in RPMI culture medium (1.0 g/L glucose), supplemented with 10% *v/v* heat-inactivated fetal bovine serum (FBS), 1% *v/v* L-glutamine and 1% *v/v* penicillin-streptomycin. Cells were cultured at 37°C with 100% humidity and 5% CO_2_, and subcultured when confluency reached 80%.

### Generation of Parp7 knockout cells

The guide oligos used to make the gRNA were: 5´- CACCGTCTTCTCAGAAATTCTCATT-3´ and 5´-AAACAATGAGAATTTCTGAGAAGAC-3´. After inserting the resulting gRNA into the PX459 plasmid, the cells were transfected, selected and expanded as previously described (32). To confirm knockout, genomic DNA from several clones was harvested, and the target site was amplified and sequenced. The primers used for sequencing were: Forward 5´-TGCAGATTTTTGCATAGCTTTTG-3´ and reverse 5´-TTGTCTTGGAAAGCTC CTGGT-3´. After screening, one clone was subsequently expanded, and further analysed. Cells transfected with an empty PX459 plasmid were also selected and expanded and will be referred to as CR705^Cas9^ cells.

### Western blotting

Cells used for western blotting were seeded in six-well plates and treated the following day. Cells were lysed in TE-buffer supplemented with 1% *w/v* SDS. After brief sonication, the samples were boiled at 95°C for 10 min. Protein concentration was determined with a BCA assay (Thermo Fisher Scientific, Waltham, MA, USA). Proteins were separated by SDS-PAGE and transferred to polyvinylidene fluoride (PVDF) membranes. The antibodies used were: lab generated anti-PARP7 antibody (35), anti-AHR (Enzo Life Sciences, Farmingdale, NY, USA; bml-sa210-0100), anti-STING (Cell Signalling Technology, Danvers, MA, USA; D2P2F), anti-STAT1 (Cell Signalling Technology; #9172), anti-STAT2 (Cell Signalling Technology; D9J7L), anti-IRF9 (Cell Signalling Technology; D9I5H), anti-pSTAT1 (Y701) (Cell Signalling Technology; D4A7), and anti-β-actin (Sigma-Aldrich; AC-74). After incubation with corresponding secondary antibody (Rabbit, Mouse, Cell Signalling Technology), the protein bands were visualized with SuperSignal™ West Dura Extended Duration Substrate or SuperSignal™ West Atto Ultimate Sensitivity Substrate (Thermo Fisher Scientific, Waltham, MA, USA).

### Real time qPCR

Total RNA was isolated using the Aurum™ Total RNA isolation kit (BioRad, Hercules, CA, USA), and used to synthesize cDNA with the High-Capacity cDNA Reverse Transcription Kit (Applied Biosystems, Waltham, MA, USA). The RT-qPCR assays were set up as previously described (35). The primers used are provided in **Supplementary Table S1**.

### Proliferation assays

Cells were seeded in 96-well plates on day 0, 24 h later the cells were treated with DMSO or 100 nM RBN-2397. Cell proliferation was measured using CellTiter Glo (Promega, Madison, WI, USA) assay according to the manufacturer’s instructions. Data are shown as a percent cell proliferation compared with 72 h DMSO treatment.

### Mouse models and tumour studies

Female immune deficient (NOD.Cg-*Prkdc^scid^ Il2rg^tm1Wjl^/SzJ*, NSG: #005557) and immunocompetent (C57BL/6J: #000664) mice were purchased from The Jackson Laboratory (Bar Harbor, ME, USA). The generation of *Parp7^H532A/H532A^* (*Parp7^HA/HA^*) mice has been described previously (29). Mice aged 8-12 weeks underwent isoflurane anaesthesia before a single subcutaneous injection with either CR705^Cas9^ or CR705^Parp7KO^ cells. CR705 cells were prepared as single cell suspensions and injected at a density of 5 × 10^5^ cells. Tumour growth was monitored with caliper measurements, and tumour volumes were calculated using the standard formula π/6 × W^2^ × L. Mice were euthanized at the end of experiments by cervical dislocation, and tumours were prepared for histological analyses. All experimental animals were housed in the Division of Comparative Medicine at the University of Toronto, with a 12 h light/dark cycle, and access to chow and water *ad libitum*. Care and treatment of animals followed the guidelines set by the Canadian Council on Animal Care and was approved by the University of Toronto Animal Care Committee.

### Immunohistochemistry

Sectioning and staining of the tumours were performed according to standard methods. Fixed tissues were provided to the HistoCore Facility at the Princess Margaret Cancer Centre (Toronto, Ontario, Canada), and sample processing, staining with Ki67, CD3, and CD8 and scanning were done at the facility. Quantification analysis was performed with QuPath v0.4.3 (36).

### RNA sequencing and data analysis

Total RNA was isolated from approximately 100 mg of tumours from CR705^Cas9^ or CR705^Parp7KO^ cells using the Aurum™ Total RNA isolation kit (BioRad) according to the manufacturer’s protocol. The raw RNA sequence paired-end fastq files were quantified using the Salmon tool with “libtype” flag as automatic and mm10 version of the Salmon index file (37). The index was generated using the salmon “index” flag with the mm10 transcripts fasta file supplied. The “tximport” import function from the tximport package [v1.26.1 (38)] was used to import the Salmon quantification data for further processing including differential expression analysis by DESeq2 (39). For all comparisons, empty vector (Cas9) tumour samples were considered as the control. Significant genes were considered as those with absolute log fold change greater than 1 and Benjamin Hochberg false discovery rate value of differential expression less than 0.01 and tested using the Wald Test implemented in DESeq2. Pathway analysis was done using the Reactome database and Ingenuity pathway analysis (Qiagen, Hilden, Germany).

### Tumour and spleen dissociation into single cells

Tumours were dissected at endpoint and were processed into single-cell suspensions using a Mouse Tumour Dissociation Kit (Miltenyi Biotec, Bergisch Gladbach, Germany) according to the manufacturer’s protocol. Approximately, 1-2 mm tumour pieces were placed in an ice-cold Tissue Storage Solution before being transferred into gentleMACS™ C Tubes (Miltenyi Biotec) containing the appropriate kit reagents and enzymes. The tumour samples were then incubated in a gentleMACS Octo Dissociator (Miltenyi Biotec). SmartStrainers were used to filter debris and erythrocyte lysis was done using Red Blood Cell Lysis Solution (Miltenyi Biotec). Cells were counted and frozen in MACS Freezing Solution and store at -80°C.

### Spectral flow cytometry analysis

Spectral flow cytometry analysis of stained cell suspensions was performed on a Cytek Aurora spectral flow cytometer equipped with a 3-laser, 38-detector array and operated by the Cytek SpectroFlo software version 3.2.1. Healthy splenocytes or Ultra-Comp control compensation particles (Thermo Fisher Scientific) defined spectral unmixing standards data for single colour controls for each of the 28 targets analysed (**Supplementary Table S2**). Cells suspensions were washed with PBS before viability staining with Zombie NIR viability dye for 15 min at room temperature. Cells were then incubated with Fc block for 15 min on ice. Staining with Anti-CD62L, TCRγδ, and CCR6 antibodies was then performed by incubating cells with optimized antibody concentrations for 30 min at 37°C. Staining with antibodies against other cell surface receptors was then performed for 30 min at room temperature. For intracellular staining, cells were fixed and permeabilized with the FoxP3 Fix/Perm buffer set (eBioscience) before incubating with anti-FoxP3 and RORγt antibodies for 1 hour at room temperature. All staining steps with fluorescent reagents were performed in the dark. The data was analysed with FlowJo v10 software.

### Statistical analysis

Data are represented as standard error of the mean (S.E.M) of at least three individual experiments and were analysed with GraphPad Prism v8.2 (San Diego, CA, USA). Statistical analyses were done using a two-tailed student’s t-test, one- or two-way analysis of variance (ANOVA). For flow cytometry, cell counts, and population frequencies were determined using FlowJo V10 software. Differences in population frequencies of different tumour infiltrating leukocyte (TIL) populations between CR705^Cas9^ and CR705^Parp7KO^ tumours were compared using Mann-Whitney test or mixed-effects analysis with Šídák’s multiple comparisons test.

## Results

### PARP7 inhibition differentially affects AHR and IFN-I signalling in PDAC cell lines

**Figure 1:**
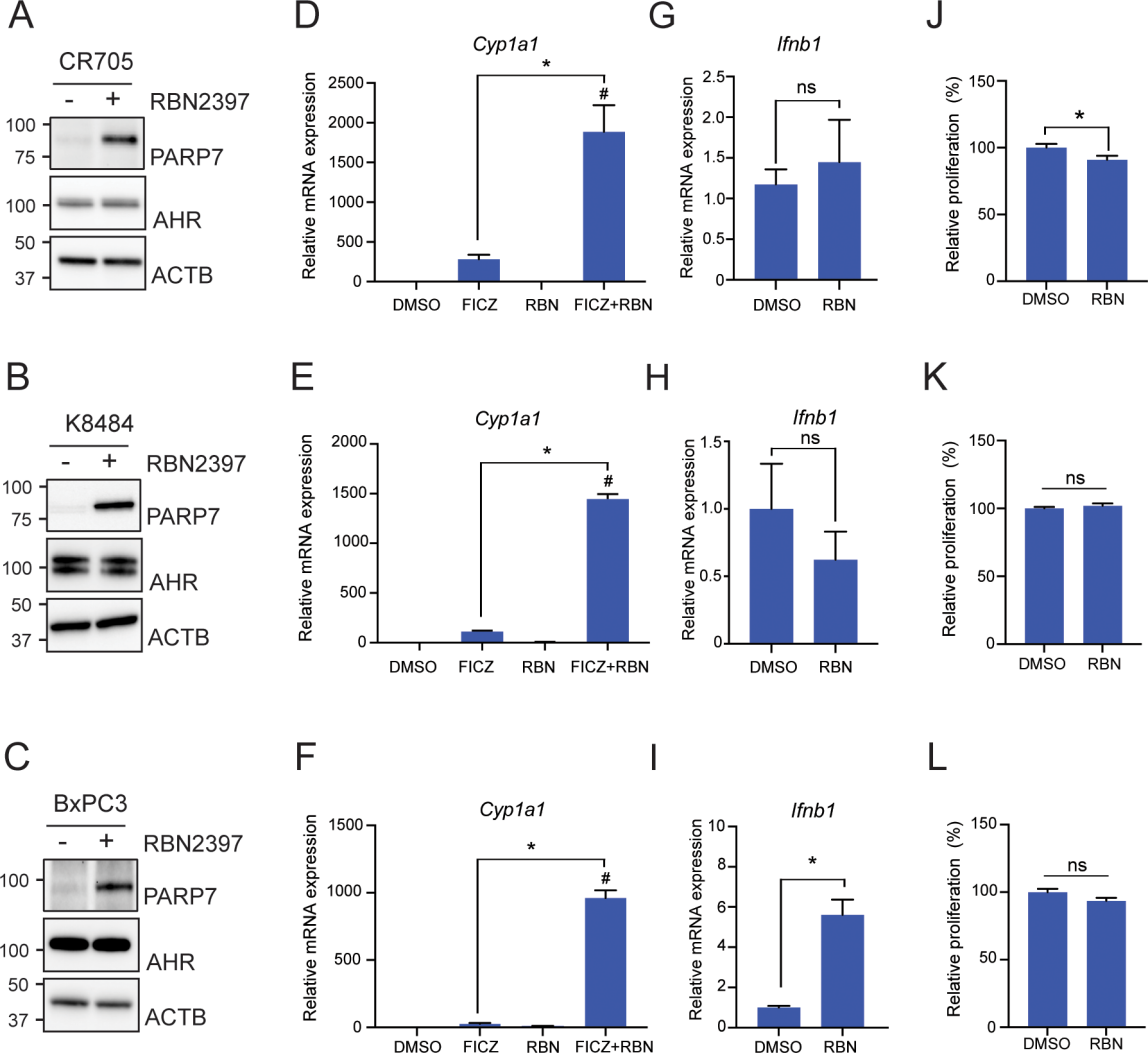
PARP7 inhibition differentially affects AHR and IFN-I signalling in CR705, K8484 and BxPC3 cells. Relative PARP7 and AHR protein levels in **(A)** CR705, **(B)** K8484 and **(C)** BxPC3 cells treated with or without RBN-2397 (100 nM) treatment for 24h. *Cyp1a1* levels are increased with PARP7i in all cell lines. **(D)** CR705, **(E)** K8484 and **(F)** BxPC3 cells were treated with 10 nM FICZ, 100 nM RBN-2397 and FICZ+RBN-2397 for 4h. RBN-2397 treatment differentially induced *Ifnb* levels in **(G)** CR705, **(H)** K8484 and **(I)** BxPC3 cells. PARP7i resulted in decreased cell proliferation of **(J)** CR705 but not **(K)** K8484 nor (**L**) BxPC3 cells. Cells were treated with 100 nM of RBN-2397 for 72h. *p<0.05 significance compared with DMSO, ^#^ *p*<0.05 significance compared with FICZ. ns, not significant.

To characterize the effects of PARP7 inhibition (PARPi) on AHR and IFN-I signalling and proliferation we exposed CR705, K8484 and BxPC3 cells to the AHR agonist, FICZ, and/or the PARP7 inhibitor, RBN-2397. CR705 and K8484 cells are PDAC cells derived from a pancreatic tumour in the *LSL-Kras^G12D/+^;LSL-Trp53^R172H/+^;Pdx1-Cre* (KPC) mouse model (34, 40). This model is commonly used to study human pancreatic cancer, as the mice spontaneously develop pancreatic cancer at 11-12 weeks of age, and the tumours display many of the key features of the TME observed in human patients (41). BxPC3 cells are a human PDAC cell line. We previously reported that PARP7 catalyses its own proteolytic degradation and that inhibition of PARP7 activity stabilizes its protein levels (35). As such, treatment with the PARP7 inhibitor RBN-2397 enabled visualization of PARP7 protein. Because of the well-established feedback inhibition that PARP7 has on AHR signalling, we also determined AHR levels in all cell lines. Western blotting confirmed PARP7 and AHR protein expression in all three cell lines (**Figures 1A–C**). The two distinct AHR protein bands in K8484 cells are consistent of the mixed 129/B6 background of the cells, since 129 mice express the long *Ahr^d^* allele (104 kDa) while C57BL/6 express the shorter *Ahr^b1^* (96 kDa) allele (42). In line with PARP7’s role as a negative regulator of AHR, 4 h co-treatment with 100 nM RBN-2397 and 10 nM FICZ significantly increased expression levels of the AHR target gene, *cytochrome P450 1a1* (*Cyp1a1*) compared with FICZ alone in all cell lines (**Figures 1D–F**). Despite PARP7’s role as an inhibitor of IFN-I signalling, 4 h treatment RBN-2397 only induced *Ifnb* levels in BxPC3 cells. We next determined whether the cell lines were sensitive to the antiproliferative actions of RBN-2397, which has been reported to be dependent on AHR expression (**Figures 1G–I**). RBN-2397 resulted in a slight (<9% decrease), albeit significant, decrease the proliferation of CR705 cells. K8484 and BxPC3 cells were insensitive to the antiproliferative effects of RBN-2397 (**Figures 1J–L**).

### Loss of PARP7 increases levels of ISGF3 and downstream signalling

**Figure 2:**
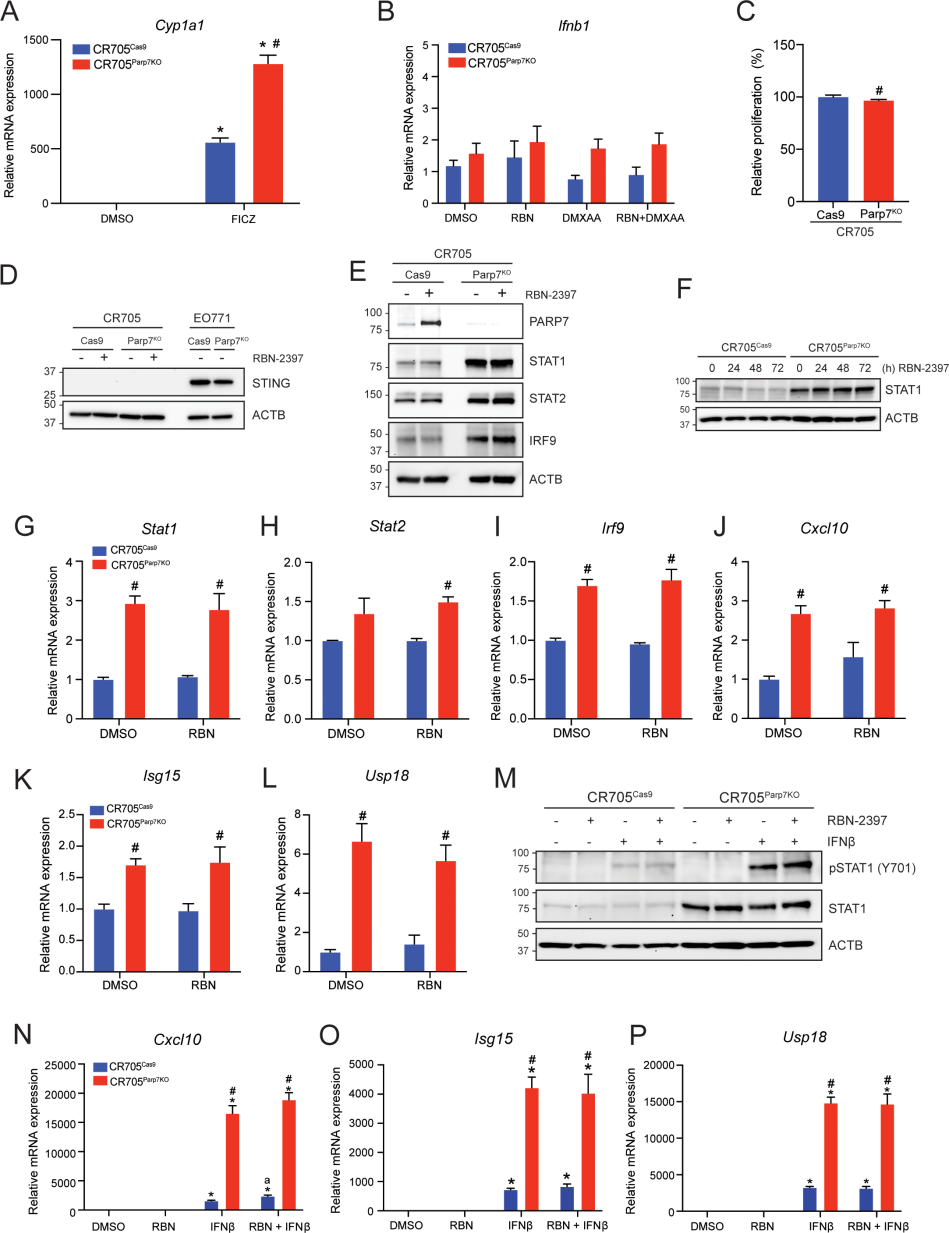
CR705^Parp7KO^ cells do not express STING but have increased levels of ISGF3 and selected ISGs. **(A)** FICZ-induced *Cyp1a1* levels were increased in CR705^Parp7KO^ compared with CR705^Cas9^ cells. Cells were treated with 10 nM FICZ for 4h. **(B)** Expression levels of *Ifnb1* did not significantly differ across the samples tested. CR705^Cas9^ and CR705^Parp7KO^ cells were treated with 100 nM RBN-2397 for 24 h and/or 10 µg/mL DMXAA for 4 h, and expression levels were determined by RT-qPCR. **(C)** Loss of *Parp7* cause a slight, but significant decrease in the proliferation of CR705 cells after 72h. **(D)** CR705 cells did not express STING. CR705 and EO771 cells were treated with 100 nM RBN-2397 for 24 h and their STING levels detected by western blotting. **(E)** Inhibition of PARP7 with 100 nM RBN-2397 for 24 h enabled visualization of PARP7 in CR705^Cas9^ cells. Protein levels of STAT1, STAT2 and IRF9 were increased in CR705^Parp7KO^ cells. **(F)** STAT1 protein levels after treatment with 100 nM RBN-2397 for 24, 48 and 72h. **(G-I)** Expression of *Stat1, Stat2* and *Irf9* mRNA levels and **(J-L)** the ISGF3 target genes *Cxcl10*, *Isg15* and *Usp18* after 24 h treatment with 100 nM RBN-2397 in control and Parp7^KO^ cells. **(M)** Treatment with 1000 U/mL IFNβ for 1 h induced phosphorylation of STAT1. **(N)** Expression levels of *Cxcl10* were significantly elevated in response to IFNβ and was further increased in CR705^Parp7KO^ and CR705^Cas9^ cells treated with RBN-2397. **(O, P)** Expression levels of *Isg15* and *Usp18* were significantly elevated in response to IFNβ, and this was further increased in the CR705^Parp7KO^ cells. Cells were treated with 100 nM RBN-2397 for 24 h and exposed to 1000 U/mL of IFNβ for 4 h **(L-N)**. *p<0.05 compared with DMSO, ^#^ *p*<0.05 significance due to Parp7 deficiency, and ^a^ *p*<0.05 significance due to PARP7 inhibition compared with IFNβ treatment alone.

Since the antitumour effects of PARP7i are reported to be dependent on IFN-I signalling (33, 43, 44), we further characterized the IFN-I pathway in CR705 cells. We chose CR705 cells because that were slightly sensitive to the antiproliferative effects of RBN-2397 and that are derived from C57BL/6 mice allowing us to do syngeneic tumour studies in immunocompetent mice. To this end we generated these *Parp7* knockout cells, using CRISPR/Cas9 gene editing. After selection and sequencing of several potential clones, only one clone contained indels that resulted in frameshift mutations in the *Parp7* gene (**Supplementary Figure S1**). This clone, referred to as CR705^Parp7KO^, was expanded and further characterized. Loss of PARP7 increased FICZ-induced increases in *Cyp1a1* levels compared with CR705^Cas9^ control cells (**Figure 2A**). We then tested whether *Parp7* loss affect IFN-I signalling. Cells were treated with RBN-2397 and co-treated with the murine specific STING agonist 5,6-dimethylxanthenone-4-acetic acid (DMXAA) (**Figure 2B**). *Ifnb1* levels were unaffected by RBN-2397 and DMXAA alone or by their co-treatment. *Ifnb1* levels did not significantly differ between CR705^Cas9^ and CR705^Parp7KO^ cells. CR705^Parp7KO^ cells proliferated slightly, but significantly, less (< 5%) than CR705^Cas9^ cells (**Figure 2C**), suggesting that they are weakly sensitive to the anti-proliferative effects following *Parp7* loss.

Consistent with the lack of inducible *Ifnb* levels, neither CR705^Cas9^ nor CR705^Parp7KO^ cells expressed STING (**Figure 2D**). STING expressing EO771^Cas9^ and EO771^Parp7KO^ cells were used as positive controls. Western blots also confirmed that lack of PARP7 levels in CR705^Parp7KO^ cells even after RBN-2397 treatment (**Figure 2E**). Since we previously reported that the ISGF3 complex was upregulated in *Parp7* deficient cells, we tested whether this was also observed in CR705 cells (**Figure 2E**) (32). As expected, we observed that PARP7 protein levels were stabilized after treatment with RBN-2397. In agreement with previous findings, the expression levels of STAT1, STAT2 and IRF9, which form ISGF3, were upregulated in the CR705^Parp7KO^ cells, but not in CR705^Cas9^ cells treated with RBN-2397. We were unable to detect phosphorylation of STAT1 in these samples (data not shown). In contrast to previous observations in EO771 cells, longer exposure to RBN-2397 (48 and 72 h) did not increase STAT1 levels in control cells (**Figure 2F**) (32). In line with the elevated protein levels, we also observed significant increases in *Stat1* and *Irf9* mRNA levels, but not *Stat2* mRNA levels in CR705^Parp7KO^ cells (**Figures 2G–I**).

We then determined the expression levels of ISGF3 target gene *Cxcl10* to evaluate the downstream signalling pathway and found the levels to be significantly increased in CR705^Parp7KO^ cells (**Figure 2J**) (45). To further confirm increased ISGF3 signalling in these cells, we tested the expression level of target genes *Isg15* and *Usp18* (**Figures 2K, L**). Both *Isg15* and *Usp18* mRNA levels were significantly upregulated in CR705^Parp7KO^ cells, but not in control cells treated with RBN-2397. Longer exposure to RBN-2397 (48 and 72 h) did not further elevate the expression levels of these target genes (data not shown). Exposure to exogenously added IFNβ resulted in phosphorylation of STAT1, but this was not further increased by RBN-2397 (**Figure 2M**). CR705^Parp7KO^ cells displayed increased levels of phosphorylated STAT1, but quantification of the bands revealed that the relative levels of pSTAT1 compared to the native STAT1 were lower than in CR705^Cas9^ cells (**Supplementary Figure S2A**). Downstream target genes *Cxcl10*, *Isg15* and *Usp18* were all significantly increased in response to IFNβ, and further elevated in the Parp7^KO^ cells (**Figures 2N–P**). Treatment with RBN-2397 slightly increased levels of *Cxcl10* in control cells but did not affect the levels of *Isg15* or *Usp18* (**Supplementary Figures S2B-D**). Taken together, these findings indicate that CR705 cells exhibit a partially functional IFN-I signalling pathway with increased responsiveness after *Parp7* loss. Furthermore, exposure to IFNβ revealed an intact pathway downstream of the IFNAR complex, with increased activity in the *Parp7* deficient cells. This may implicate a role for PARP7 in IFN-I signalling that is independent of upstream secretion of IFNβ.

### Loss of *Parp7* affects expression of ARTD family members

**Figure 3:**
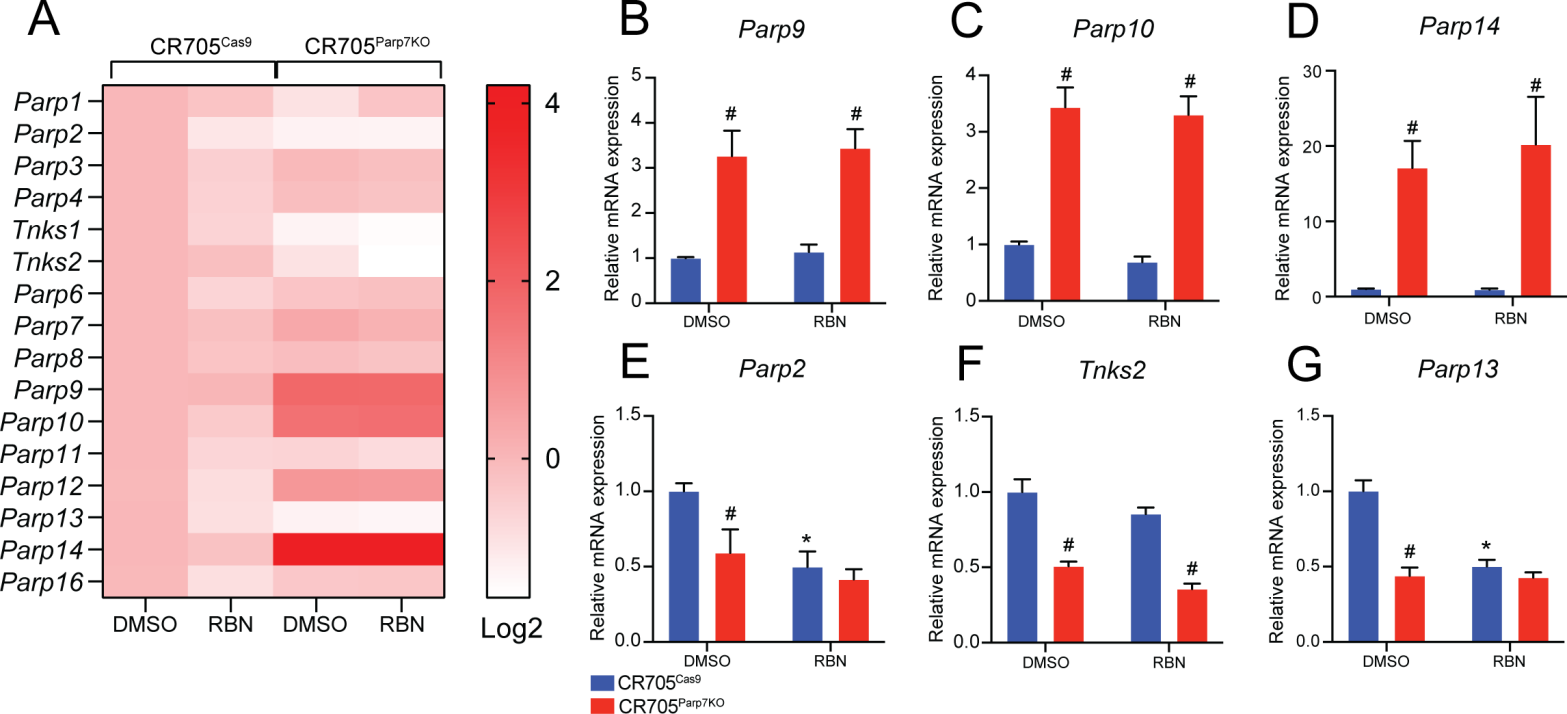
Loss of *Parp7* affects expression levels of ARTD family members. **(A)** Heatmap showing expression levels of ARTD family members in CR705^Cas9^ and CR705^Parp7KO^ cells after RBN-2397 treatment for 24h. The data are presented as log2-fold changes compared to the DMSO-treated control cells. **(B-D)** Levels of *Parp9*, *Parp10* and *Parp14* were significantly increased in the CR705^Parp7KO^ cells, but unaffected by RBN-2397. **(E-G)** Levels of *Parp2*, *Tnks2* and *Parp13* were significantly lower in CR705^Parp7KO^ cells. *Parp2* and *Parp13* were decreased after PARP7 inhibition in the control cells. * denotes statistical significance (*p*<0.05) from the DMSO treated samples, while ^#^ denotes significance (*p*<0.05) due to loss of *Parp7*.

Because *Parp7* loss has been reported to resulted in increased expression of several members of the ARTD family (32), we determined their expression levels in CR705^Cas9^ and CR705^Parp7KO^ cells (**Figure 3A**). Consistent with our previous findings, the levels of the MARylating enzymes *Parp9*, *Parp10* and *Parp14* were significantly upregulated in CR705^Parp7KO^ cells, but not in CR705^Cas9^ cells after treatment with RBN-2397 (**Figures 3B–D**). Notably, PARP14 is also a target gene and positive regulator of the IFN-I pathway (46). Both loss and inhibition of PARP7 resulted in decreased expression of *Parp2* and *Parp13* (**Figures 3E, G**), while *Parp7* loss, but not inhibition, decreased levels of *Tnks2* (**Figure 3F**). The individual graphs for the other PARPs are provided in **Supplementary Figure S3**.

### Loss of *Parp7* in CR705 PDAC cells reduces tumour growth

**Figure 4:**
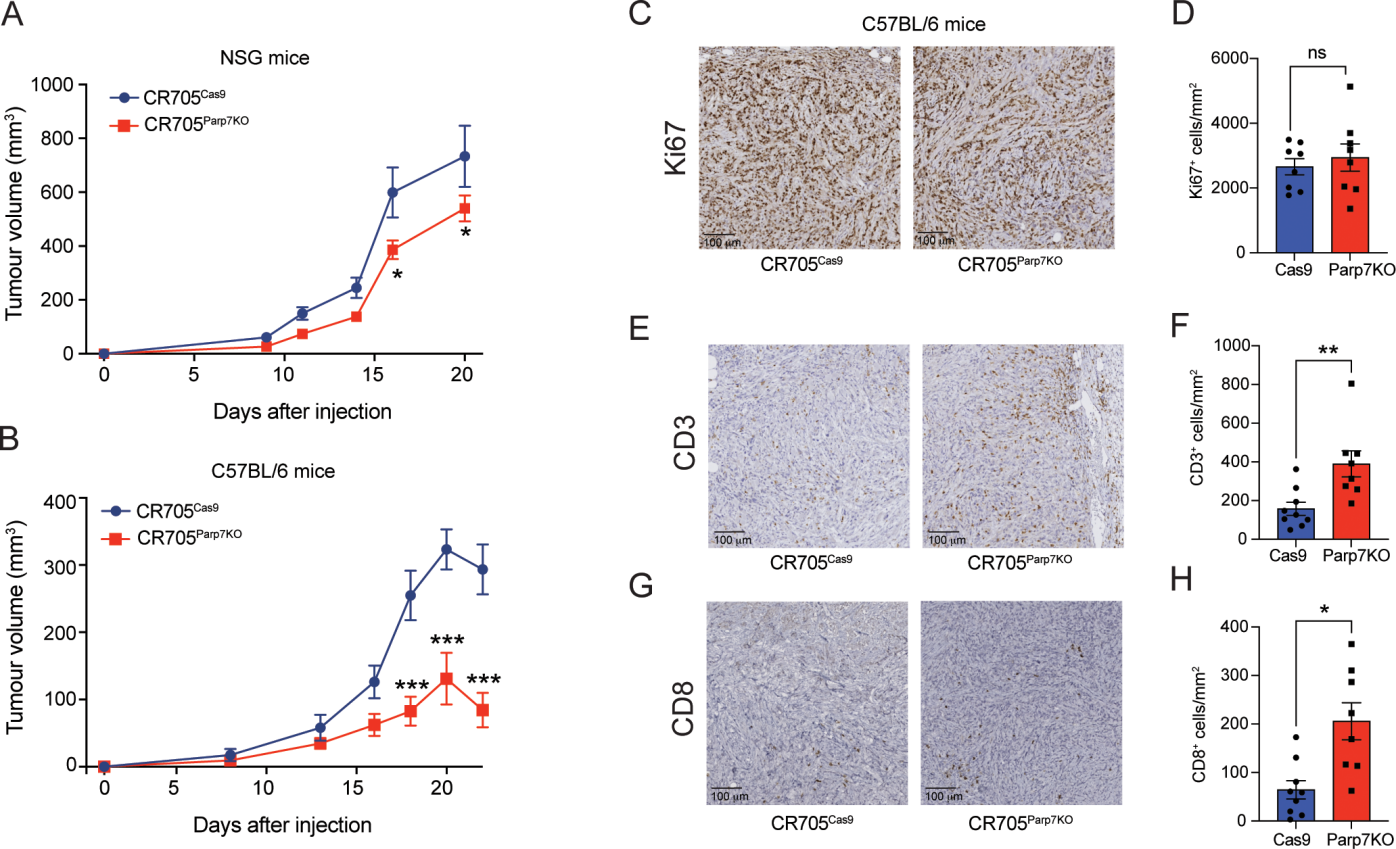
CR705^Parp7KO^ cells form smaller tumours when injected into immunocompetent mice. **(A)** CR705^Parp7KO^ cells gave rise to smaller tumors when injected into immunodeficient NSG mice. *n*=6. **(B)** CR705^Parp7KO^ cells gave rise to smaller tumours when injected into right flank of female C57BL/6 mice. *n*=8-9. **(C)** Representative images of Ki67 staining. **(D)** Quantification of Ki67^+^ cells, displayed as positive cells per mm^2^. **(E)** Representative images of CD3 staining. **(F)** CR705^Parp7KO^ tumours had increased levels of infiltrating CD3^+^ cells compared with CR705^Cas9^ tumours. Quantification of CD3^+^ cells, displayed as positive cells per mm^2^. **(G)** Representative images of CD8 staining. **(H)** CR705^Parp7KO^ tumours had increased levels of infiltrating CD8^+^ cells compared with control. *p<0.05, ** *p*<0.001, ***p<0.0001. ns, not significant.

To determine the role of PARP7 on the ability of CR705 to form tumours *in vivo*, we injected CR705^Cas9^ and CR705^Parp7KO^ cells into immune deficient NSG mice. Injection of CR705^Parp7KO^ cells gave raise to tumours that grew more slowly compared with CR705^Cas9^ cells with an average final CR705^Parp7KO^ tumour volume of 73% that of CR705^Cas9^ tumours. (**Figure 4A**). Because IFN-I signalling was increased in CR705^Parp7KO^ cells and it is known to enhance immune cell-mediated anti-tumour effects, we next tested the abilities of CR705^Cas9^ and CR705^Parp7KO^ cells to form tumours in immunocompetent C57BL/6 mice. Injection of CR705^Parp7KO^ cells gave rise to smaller tumours compared with injection of CR705^Cas9^ cells in C57BL/6 mice (**Figure 4B**) and this effect was greater than that observed in NSG mice with final CR705^Parp7KO^ tumour volume of 33% that of CR705^Cas9^ tumours. We did not observe any differences in the number Ki67^+^ cells, a marker of cell proliferation from CR705^Cas9^ or CR705^Parp7KO^ tumours in C57BL/6 mice (**Figure 4C, D**). However, increased staining of T cells (CD3^+^) and specifically CD8^+^ T cells was observed in CR705^Parp7KO^ compared with CR705^Cas9^ tumours (**Figures 4E–H**).

### Tumours deficient in *Parp7* display a less immunosuppressive repertoire of infiltrating immune cells

**Figure 5:**
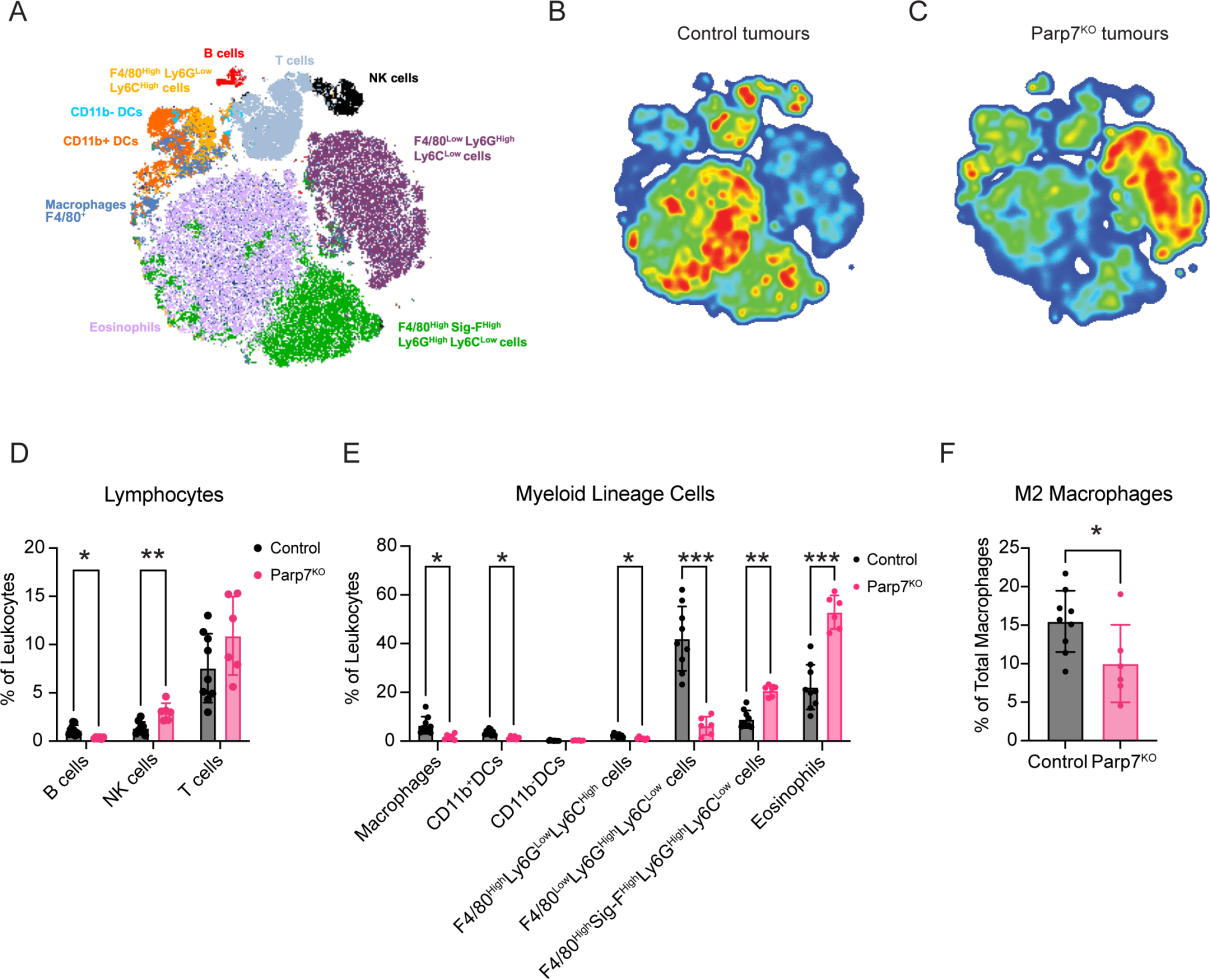
Spectral flow cytometry identifies differences in the proportion of tumour infiltrating leukocytes (TILs) between CR705^Cas9^ and CR705^Parp7KO^ tumours. **(A)** t-stochastic neighbour embedding (t-SNE) plot of TIL cell populations. Density plots of TIL cell populations from **(B)** CR705^Cas9^ and **(C)** CR705^Parp7KO^ tumours. Red is higher density whereas blue is lower density. Changes in tumour infiltrating **(D)** lymphocytes and **(E)** myeloid lineage cells as a percentage of total leukocytes in CR705^Cas9^ and CR705^Parp7KO^ tumours. **(F)** Changes in M2 macrophages as a percentage of total macrophages in CR705^Cas9^ and CR705^Parp7KO^ tumours. Cell counts and population frequencies determined using FlowJo V10 software. Differences in population frequencies of different TIL populations between CR705^Cas9^ and CR705^Parp7KO^ tumours were compared using Mann-Whitney test or mixed-effects analysis with Šídák’s multiple comparisons test. *p<0.05, **p<0.01, ***p<0.001.

Spectral flow cytometry was then used to assess differences in the proportion of tumour infiltrating leukocyte (TIL) populations between CR705^Parp7KO^ and CR705^Cas9^ tumours. A broad range of TILs including several lymphocyte, and myeloid lineage cell populations were identified based on their expression of characteristic cell surface markers (**Figures 5A–C**; **Supplementary Figure S4**). Relative proportions of each population were reported as a percentage of the total CD45^+^ leukocytes in the tumour. TILs from CR705^Parp7KO^ tumours had a higher proportion of NK cells, eosinophils, and F4/80^High^ Sig-F^High^ Ly6G^High^ Ly6C^Low^ cells. This latter population could be inclusive of mature and long-lived neutrophils (47) and myeloid-derived suppressor cells (MDSCs) (48) (**Figures 5D, E**). In contrast, CR705^Parp7KO^ tumours had a lower proportion of B cells, macrophages, and CD11b^+^ dendritic cells (CD11b^+^ DCs). Although we observed a continuum of macrophage phenotypes rather than distinct populations, we were able to use staining controls to delineate M1-like (CD80^High^ CD206^Low^) pro-inflammatory and M2-like (CD80^Low^ CD206^High^) anti-inflammatory cells (**Supplementary Figure S5A**). A similar proportion of macrophages from CR705^Parp7KO^ and CR705^Cas9^ tumours had an M1 phenotype (**Supplementary Figure S5B**); however, a lower proportion with an M2 phenotype were observed specifically in CR705^Parp7KO^ tumours (**Figure 5F**). The net difference in relative macrophage abundance was absorbed by populations we could not identify as either M1 or M2 (**Supplementary Figure S5A**). Further, CR705^Parp7KO^ tumours harboured fewer F4/80^Low^ Ly-6G^High^ Ly-6C^Low^ and F4/80^High^ Ly-6G^Low^ Ly-6C^High^ cells, which have been previously defined as polymorphonuclear and monocytic MDSCs, respectively. No differences in the expression of the immune checkpoint PD-L1 were observed across all TIL populations (**Supplementary Figure S5C**).

**Figure 6:**
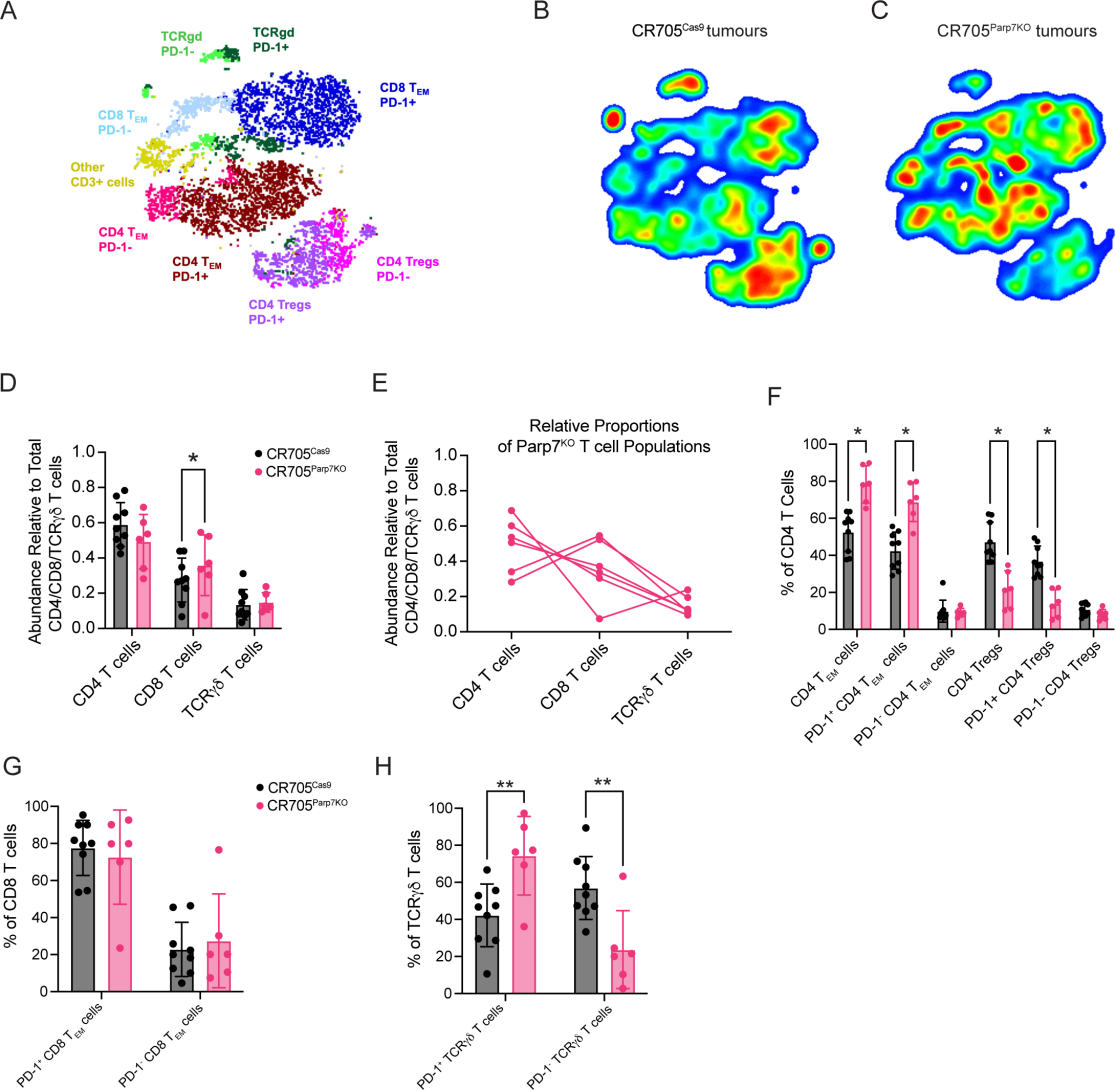
Spectral flow cytometry identifies differences in the proportion of tumour infiltrating T cell populations between CR705^Cas9^ and CR705^Parp7KO^ tumours. **(A)** t-stochastic neighbour embedding (t-SNE) plot of T cell populations. Density plots of T cell populations from **(B)** CR705^Cas9^ and **(C)** CR705^Parp7KO^ tumours. Red is higher density whereas blue is lower density. **(D)** Relative abundance of T cell populations in CR705^Parp7KO^ compared with CR705^Cas9^ tumours. **(E)** Relative proportions of CD4, CD8 and TCRγδ relative to T cells in CR705^Parp7KO^ tumours. Changes in the percentages of **(F)** CD4, **(G)** CD8 and **(H)** TCRγδ subsets between CR705^Parp7KO^ compared with CR705^Cas9^ tumours. Cell counts and population frequencies determined using FlowJo V10 software. Differences in population frequencies between CR705^Cas9^ and CR705^Parp7KO^ tumours were compared using Mann-Whitney test or mixed-effects analysis with Šídák’s multiple comparisons test. *p<0.05, **p<0.01.

Despite the average proportion of total T cells from CR705^Parp7KO^ tumours being slightly higher than that of CR705^Cas9^ tumours, this was not statistically significant (**Figure 5D**). To better understand the effect of *Parp7* loss on T cell tumour infiltration, we also used a dedicated spectral flow cytometry panel capable of differentiating cancer-relevant T cell subpopulations including cytotoxic (CD8), helper (CD4), and TCRγδ T cells (**Figures 6A–C**; **Supplementary Figure S6**). We next determined the proportion of each of these major T cell populations, reported as their abundance relative to the total number of CD4^+^, CD8^+^, and TCRγδ T cells. A higher proportion of cytotoxic CD8^+^ T cells was observed in CR705^Parp7KO^ tumours relative to CD4^+^ and TCRγδ T cells (**Figure 6D**). Although the difference in the proportion of CD4^+^ T cells was not statistically significant, CR705^Parp7KO^ tumours with the highest relative CD8^+^ T cell infiltration also had the lowest relative CD4^+^ T cell infiltration (**Figure 6E**). Within the CD4^+^ T cell compartment, there were more T effector/memory (T_EM_; CD44^+^, CD62L^-^) and less T regulatory (Treg; FoxP3^+^) cells in CR705^Parp7KO^ compared with CR705^Cas9^ tumours (**Figure 6F**). This was specifically reflected in the relative proportion of cells expressing high levels of the immune checkpoint protein PD-1, with no difference in the proportion of PD-1^Low^ CD4^+^ T_EM_ or Tregs. Unlike CD4^+^ T cells, there was no significant difference in the proportion of CD8^+^ T cells that were PD-1^High^ (**Figure 6G**). However, a higher proportion of TCRγδ T cells were PD-1^High^ in CR705^Parp7KO^ tumours (**Figure 6H**). Overall, these findings show that CR705^Parp7KO^ tumours had a higher proportion of cytotoxic CD8^+^ T cell infiltration, as well as a lower relative amount of PD-1^High^ CD4^+^ Tregs vs. effector memory CD4^+^ T cells. The higher ratio of effector T cells:Tregs coupled with the lower proportion of M2 macrophages and increased relative amounts of cytotoxic NK cells suggest that loss of *Parp7* may counteract the development of an immunosuppressive TME.

### Gene expression profiling of CR705^Parp7KO^ tumours reveals increased activation of interferon and other immune signalling pathways

**Figure 7:**
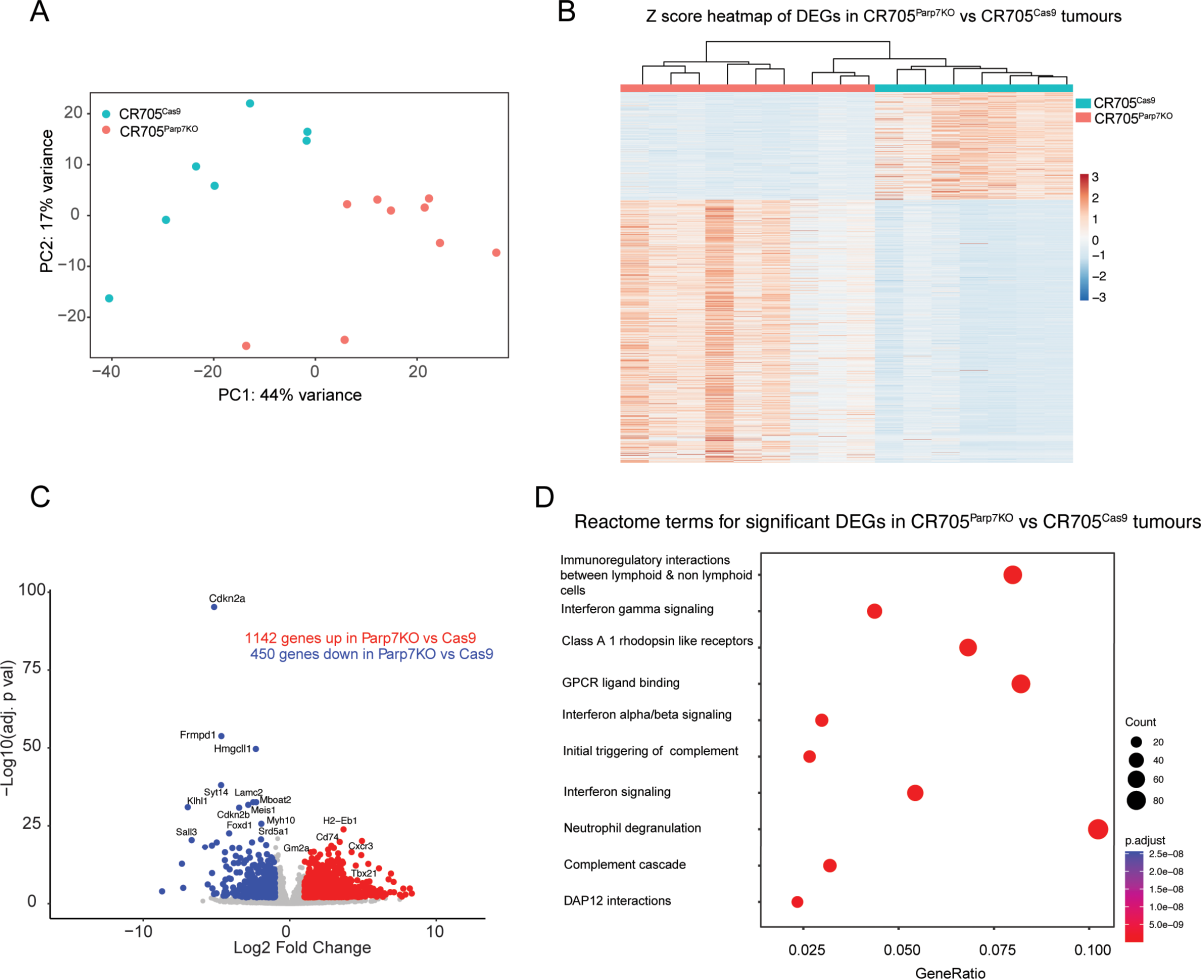
Gene expression profiling of CR705^Parp7KO^ and CR705^Cas9^ tumours reveals an increased number of genes regulating interferon and inflammatory immune signalling. **(A)** Principal component analysis (PCA) plot of all samples. **(B)** Heatmap displaying illustrating the relative gene expression profiles of overlapping differentially expressed genes in CR705^Parp7KO^ and CR705^Cas9^ tumours for each replicate. Genes and individual samples are arranged by hierarchical clustering. CR705^Parp7KO^ and CR705^Cas9^ tumours are indicated with coloured bars at the top of the heat map. **(C)** Volcano plot showing the genes that are significantly upregulated and downregulated genes. Differentially expressed genes were determined using an absolute log2 fold change 1 and an adjusted p value < 0.01. **(D)** Top 10 pathways identified using the Reactome database that were significantly changed in Parp7^KO^ compared with Cas9 tumours.

RNA sequencing was then used to investigate gene expression profiles and identify cellular pathways differentially regulated between CR705^Cas9^ and CR705^Parp7KO^ tumours. Principal component analysis revealed distinct clustering between CR705^Cas9^ and CR705^Parp7KO^ tumours (**Figure 7A**). Hierarchical clustering in the form of a heatmap was used to show distinct expression patterns for overlapping differentially expressed genes (DEGs) between CR705^Cas9^ and CR705^Parp7KO^ tumours (**Figure 7B**). We identified 1592 significantly changed genes of which 1142 were increased and 450 were decreased in CR705^Parp7KO^ vs CR705^Cas9^ tumours (**Figure 7C**; **Supplementary Table S3**). Gene expression profiling confirmed increased expression levels of other members of the ARTD family including *Parp9*, *Parp10*, *Parp12* and *Parp14* in Parp7^KO^ tumours. *Parp7* (*Tiparp*) levels were also increased in *Parp7* deficient tumours, which might be due to *Parp7* levels in TILs. *Cxcl10* and its receptor, *Cxcr3*, as well as *granzyme A* (*Gzma*) and *granzyme B* (*Gzmb*) levels, which support an activated T cell responses, were elevated in CR705^Parp7KO^ tumours (**Table 1**; **Supplementary Table S3**). Pathway analysis using the Reactome database revealed increases in many immune regulated pathways in CR705^Parp7KO^ tumours including immunoregulatory interactions, interferon signalling pathways, complement activation, neutrophil degranulation, as well as in GPCR signal pathways (**Figure 7D**). These data are consistent with PARP7’s role in regulating IFN signalling and suggest that the loss of PARP7 results in increased inflammation in the TME that may lead to increased immune infiltration and enhanced anti-tumour responses.

**Table 1:** Differentially expressed genes (DEGs) in CR705^Parp7KO^ compared with CR705^Cas9^ tumours.

Gene symbol	Fold change	p adj
T Cell markers		
*Cd8a*	6.2	0.00031
*Cd8b1*	8.2	2.1 x 10^-5^
*Gzmb*	3.6	0.00148
*Gzma*	11.5	6.5 x 10^-6^
Interferon signalling		
*Cxcl10*	5.0	6.4 x 10^-6^
*Cxcr3*	18.7	2.7 x 10^-17^
*Ifng*	4.9	0.00711

### Reduced growth of CR705^Cas9^ and CR705^Parp7KO^ tumours in *Parp7^HA/HA^* compared with C57BL/6 mice

**Figure 8:**
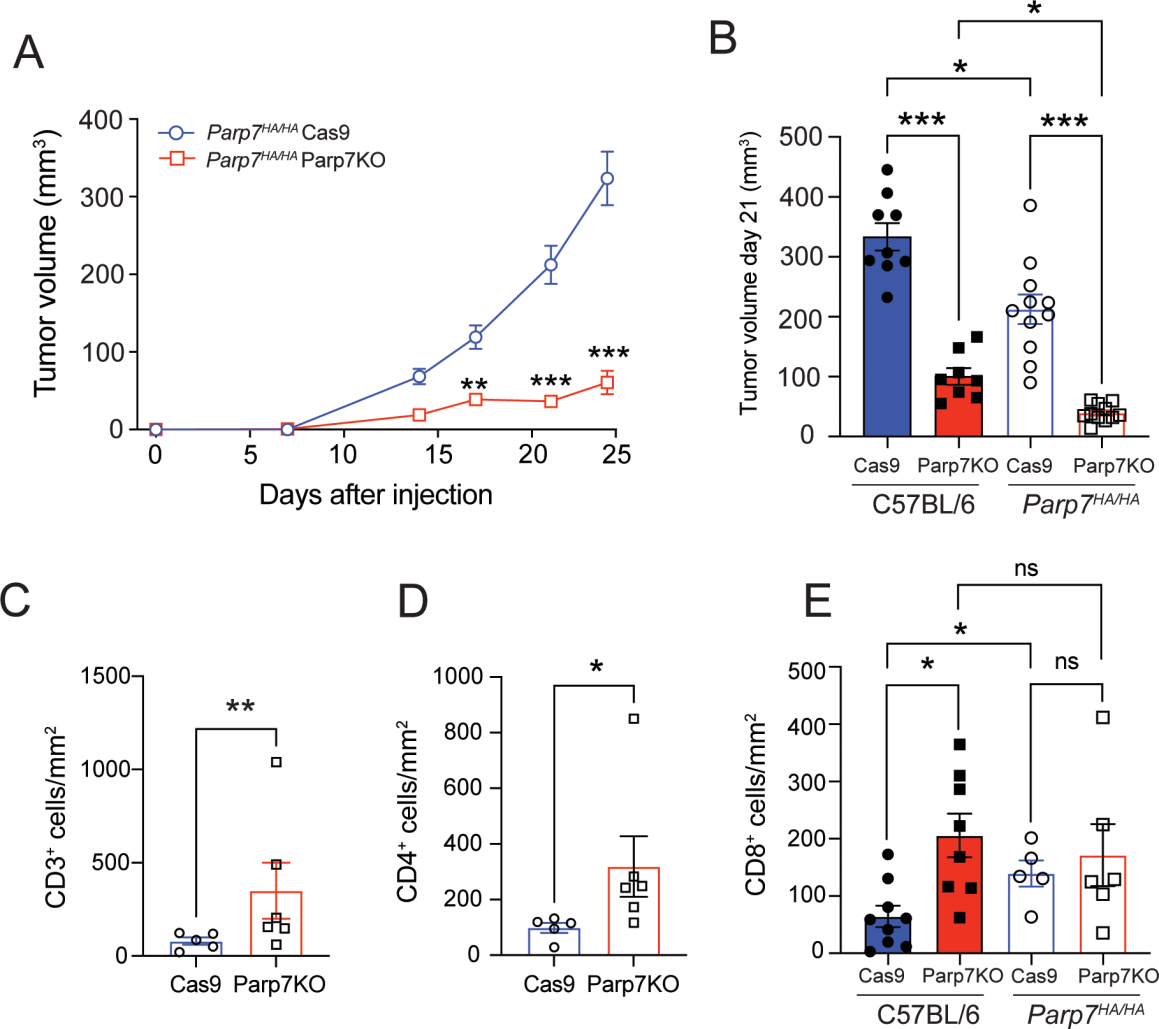
CR705^Parp7KO^ cells form smaller tumours when injected into *Parp7^HA/HA^* catalytic deficient mice. **(A)** CR705^Parp7KO^ cells gave rise to smaller tumours mice compared with CR705^Cas9^ cells when injected into female *Parp7^HA/HA^*. *n*=5-6. **(B)** At day 21 after injection, CR705^Parp7KO^ and CR705^Cas9^ tumours grew more slowly in *Parp7^HA/HA^* compared with C57BL/6 mice. *n*=8-11. **(C)** CD3^+^ T cells and **(D)** CD4^+^ T cells compared with CR705^Cas9^ tumours. **(E)** CR705^Parp7KO^ tumours had increased levels of infiltrating CD8^+^ cells compared with control. *p<0.05, ** *p*<0.001, ***p<0.0001. ns, not significant.

**Figure 9:**
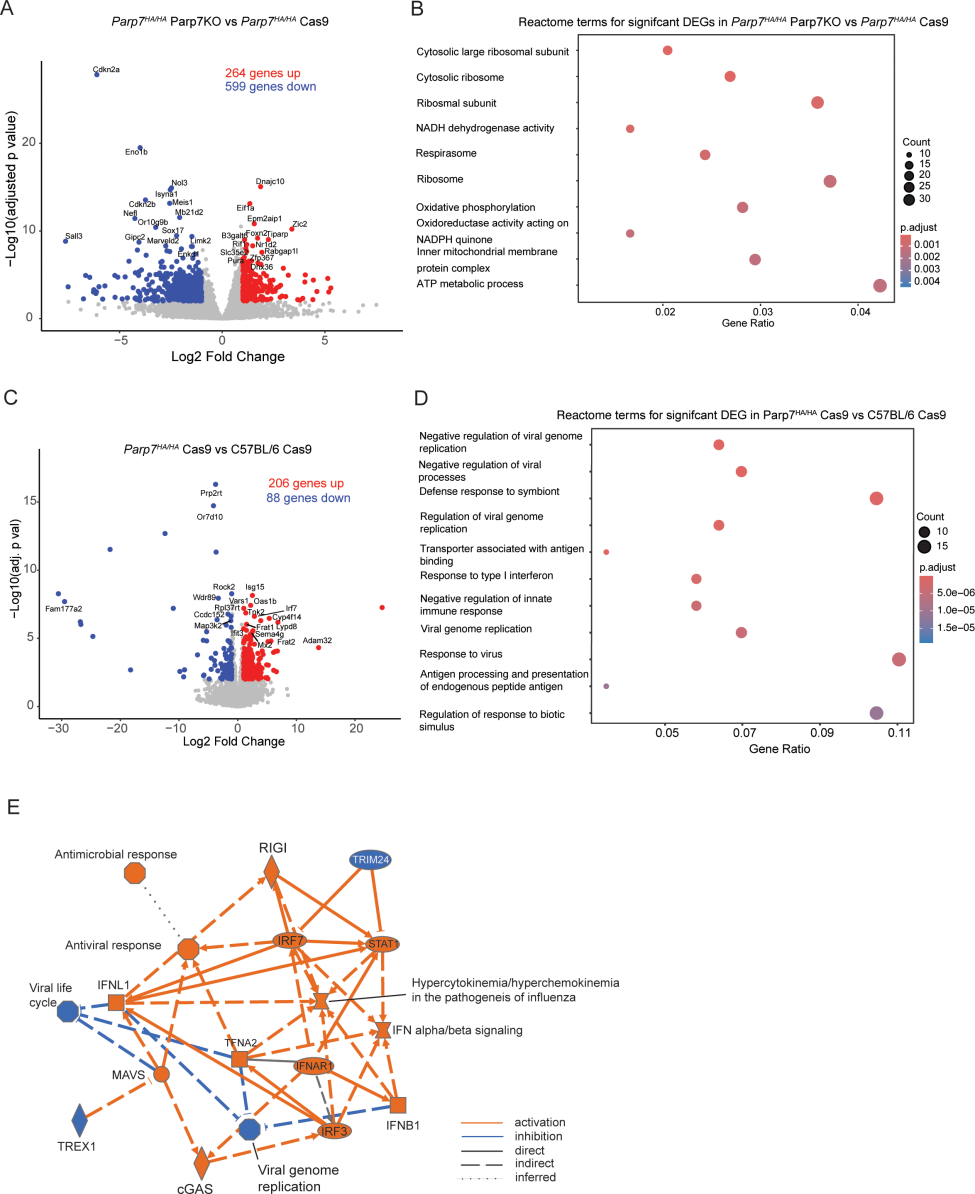
Gene expression profiling of CR705^Parp7KO^ and CR705^Cas9^ tumours in *Parp7^HA/HA^* mice. **(A)** Volcano plot showing the genes that are significantly upregulated and downregulated genes CR705^Parp7KO^ vs CR705^Cas9^ tumours in *Parp7^HA/HA^* mice. Differentially expressed genes were determined using an absolute log2 fold change 1 and an adjusted p value < 0.01. **(B)** Top 10 pathways identified using the Reactome database that were significantly changed in Parp7^KO^ compared with Cas9 tumours. **(C)** Volcano plot of significantly upregulated and downregulated genes Cas9 tumours in *Parp7^HA/HA^* compared with C57BL/6 mice. **(D)** Top 10 pathways identified using the Reactome database that were significantly changed in Cas9 tumours in *Parp7^HA/HA^* compared with C57BL/6 mice. **(E)** Graphical summary of the most significant genes or pathways and the connections among them as determined by Ingenuity pathway analysis of DEGs from Cas9 tumours *Parp7^HA/HA^* compared with C57BL/6 mice. Orange lines specify activation, blue lines specify inhibition, solid lines indicate direct interaction, dashed lines indicate indirect interaction, and dotted lines indicate an inferred relationship.

To determine if loss of PARP7 activity in immune cells and other cells in the tumour microenvironment contributes to reduced tumour growth, we injected CR705^Cas9^ and CR705^Parp7KO^ cells into *Parp7^HA/HA^* mice. *Parp7^HA/HA^* harbour a histidine 532 to alanine mutation in Parp7 resulting in a catalytic deficient PARP7. Characterisation of *Parp7^HA/HA^*, also known as *Tiparp^H532A/H532A^* mice, has been described elsewhere (29, 32). Injection of CR705^Parp7KO^ cells into *Parp7^HA/HA^* mice gave raise to tumours that grew more slowly compared with CR705^Cas9^ cells (**Figure 8A**). A comparison of the tumour volumes on day 21 between *Parp7^HA/HA^* than in C57BL/6 mice showed that CR705^Cas9^ and CR705^Parp7KO^ tumours were significantly smaller in *Parp7^HA/HA^* compared with C57BL/6 mice (**Figure 8B**). We observed increased staining of T cells (CD3^+^) and CD4^+^ in CR705^Parp7KO^ compared with CR705^Cas9^ tumours (**Figures 8C–E**). No increases in the staining of CD8^+^ T cells were observed in CR705^Parp7KO^ compared with CR705^Cas9^ tumours in *Parp7^HA/HA^* mice. However, *Parp7^HA/HA^* Cas9 tumours had increased staining of CD8^+^ T cells compared with C57BL/6 Cas9 tumours (**Figure 8E**). RNA sequencing was then used to investigate gene expression changes and identify cellular pathways that were differentially regulated between CR705^Cas9^ and CR705^Parp7KO^ tumours in *Parp7^HA/HA^* mice. We identified 863 significantly changed genes of which 264 were increased and 599 were decreased in CR705^Parp7KO^ vs CR705^Cas9^ tumours (**Figure 9A**; **Supplementary Table S4**). Pathway analysis using the Reactome database revealed enrichment in pathways involving ribosomes, cellular metabolism and oxidative phosphorylation, but not inflammatory or immune regulated pathways (**Figure 9B**).

To further investigate the role of PARP7 in IFN-I signalling in non-cancer cells of the TME, we determined gene expression changes and cellular pathways differentially regulated between CR705^Cas9^ tumours in *Parp7^HA/HA^* and C57BL/6 mice. We identified 294 DEGs of which 206 were increased and 88 were decreased in *Parp7^HA/HA^* and C57BL/6 mice (**Figure 9C**; **Supplementary Table S5**). Reactome pathway analysis revealed the enrichment of pathways involved in the negative regulation of viral replication and viral processes, and response to type I interferons (**Figure 9D**). Ingenuity pathway analysis was also used to identify cellular pathways that were commonly changed between CR705^Cas9^ tumours in *Parp7^HA/HA^* and C57BL/6 mice (**Figure 9E**). Consistent with the Reactome pathway analysis, increases in IFN-I signalling and antiviral activity were identified. These data show that loss of PARP7 activity in other cells in the TME also leads to increased IFN-I signalling resulting in reduced tumour growth.

## Discussion

PARP7i or loss of *Parp7* reduces cancer cell growth in preclinical tumour models of colon and breast cancer through increased immune cell infiltration and antitumour immunity. Antitumour activity following PARP7 inhibition is dependent on CD8^+^ T cells and enhances cancer immunotherapy in combination with anti-PD-1 immune checkpoint inhibition treatment (44). Since cancer immunotherapy has so far been unsuccessful against PDAC, we were interested to characterize PARP7-dependent IFN-I signalling and tumour growth in a model of PDAC. Using CR705 cells, a mouse PDAC cell line from a spontaneously developed pancreatic tumour in the KPC mouse, we show that loss of *Parp7* increases IFN signalling and reduces tumour growth due to increased immune cell infiltration.

Previous studies have reported that the anti-proliferative effects following PARP7 inhibition depend on multiple factors including AHR (49, 50). In agreement with these data, CR705 cells express AHR and loss or inhibition of PARP7 reduced their proliferation compared with control cells. However, neither K8484 nor BxPC3 cells which express both AHR and PARP7 were sensitive to the antiproliferative effects of PARP7i under our assay conditions. We also observed that AHR signalling was increased in *Parp7* deficient cells, which is consistent with PARP7 acting as a negative regulator of AHR (27, 28). Despite studies reporting that loss or inhibition of PARP7 enhances AHR signalling, how this crosstalk affects IFN-I signalling remains unclear (27, 29, 49). Our previous studies in EO771 cells show that PARP7 loss or inhibition increases IFN-I signalling in the absence of AHR (32). This suggests that AHR expression is not sufficient to described sensitivity to antiproliferative effects of PARP7i and that PARP7i-dependent increases in IFN-I signalling are not fully dependent on AHR.

We noted that the expression levels of *Ifnb1* in CR705 cells were lower than those observed in other cell lines, such as EO771, and they did not significantly differ between the CR705^Cas9^ and CR705^Parp7KO^ cells (32). Induction of *Ifnb1* was not further increased by treatment with the mouse-specific STING-agonist DMXAA. In line with this, we failed to detect STING expression in CR705^Cas9^ and CR705^Parp7KO^ cells. Thus, it would be intriguing to investigate whether the induction or overexpression of STING could enhance the expression of *Ifnb1*, and whether this effect would be amplified in *Parp7* deficient cells. Since downstream IFN-I signalling is mediated by ISGF3, we evaluated whether these proteins were affected by *Parp7* loss. In agreement with previous findings, we observed that all three proteins in the ISGF3 complex were upregulated in the CR705^Parp7KO^ cells (32). However, unlike our previous studies on PARP7 inhibition and knockout in EO771 cells, selected target genes of ISGF3 signalling were upregulated in response to *Parp7* loss but this was not observed in control cells after PARP7 inhibition. This could be due to adaptation to the loss of PARP7 expression or clonal selective pressures during the isolation of the CR705^Parp7KO^ clone. Despite repeated screening, we were only able to isolate one CR705^Parp7KO^ clone with indels that resulted in frameshift mutations. All other clones that were screened had indels that resulted in frameshift mutations but also losses of 3 or multiples of 3 nucleotides that resulted in the loss of one or more amino acids while maintaining the correct reading frame (data not shown). Because of this were unable to confirm these findings in a different knockout clone.

To test downstream IFNAR signalling, we exposed the cells to IFNβ. This resulted in phosphorylation of STAT1 and increased expression levels of target genes, which were substantially higher in the CR705^Parp7KO^ cells. Although the relative levels of pSTAT1 compared to native STAT1 were lower in the CR705^Parp7KO^ cells, the total amount of pSTAT1 was higher. A possible explanation for this could be that increased signalling upregulates negative regulators of the pathway, which function by repressing phosphorylation of STAT1 (51). Nonetheless, these results imply that loss of PARP7 renders the cells more sensitive to IFN-I signalling. The exact role of ISGF3 signalling in cancer is context specific, as acute signalling is associated with anti-tumour activity, while prolonged signalling promotes tumorigenesis (52). Previous studies have found that PARP7 represses the IFN-I pathway by MARylating TBK1 (30). We did not observe any differences between the empty vector control and Parp7^KO^ cells in expression levels of *Ifnb1*, but we observed clear increases in ISGF3 protein levels and downstream IFN-I signalling. This suggests that PARP7 also regulates mechanisms downstream of TBK1, which agrees with a recent study investigating PARP7 signalling in CT26 colon cancer cells (31). The mechanisms behind the elevated ISGF3 protein levels in the absence of PARP7 remain elusive and warrant further investigation to fully understand the function of PARP7 in IFN-I signalling.

Consistent with our previous studies in EO771 mouse mammary cells, *Parp7* knockout affected the expression levels of other members of the ARTD family in CR705 cells (32). We found that *Parp9*, *Parp10* and *Parp14* were upregulated in the CR705^Parp7KO^ cells. Whether this is due to off-target effects of CRISPR/Cas9, compensation due to the lack of PARP7, or loss of PARP7-dependent regulation of the expression levels of ARTD family members is unclear. PARP9 is a positive regulator of the IFN-I response, and *PARP10* is induced by IFN-Is, and involved in antiviral responses (53, 54). *PARP14* is regulated by IFN-Is, and in turn acts as a positive regulator of the response (46). Thus, it is possible that the elevated *Parp14* levels contribute to some of the phenotypes observed in CR705^Parp7KO^ cells. Furthermore, we observed decreased levels of *Parp2*, *Tnks2* and *Parp13*. TNKS2 and PARP13 are involved in antiviral immunity (55, 56). It would be interesting to examine the effects of knockdown, inhibition or induction of these proteins and assess whether this would affect the phenotypes observed in *Parp7* deficient cells.

Studies in immunocompromised NSG mice, revealed that tumours derived from CR705^Parp7KO^ cells were smaller than those from control (Cas9) cells. However, when experiments were repeated in immunocompetent mice a greater reduction in the growth of CR705^Parp7KO^ compared with CR705^Cas9^ tumours was observed. NSG mice have no mature B or T cells, with defective macrophages and NK cells as well as a reduced complement system; however, neutrophils and monocytes are detectable in peripheral blood (57). We cannot exclude that the compromised immune response in NSG mice might also contribute to the reduced CR705^Parp7KO^ tumour growth. Regardless, our tumour studies suggest that the immune system more efficiently targets the *Parp7* deficient tumours, which agrees with PARP7 acting as a negatively regulating IFN-I signalling.

In human PDAC, the hypoxic TME favours the infiltration of immunosuppressive over pro-inflammatory immune cells with increased prevalence of MDSCs, M2 macrophages, and Tregs, rather than M1 macrophages, DCs and CD4^+^ and CD8^+^ T cells (58, 59). PDACs characterized by low B and T cell infiltration and enriched in CD4^+^ Tregs are associated with a worse prognosis (60). Gene expression profiling of CR705^Parp7KO^ compared with CR705^Cas9^ tumours in C57BL/6 mice revealed increases in IFN-I and IFNγ signalling as well as other immune signalling pathways. These data show that *Parp7* deficiency in CR705 cancer cells is sufficient to increase inflammatory and immune signalling resulting in the increased tumour infiltration of immune cells and reduced tumour growth. Despite reduced growth of CR705^Parp7KO^ compared with CR705^Cas9^ tumours in *Parp7^HA/HA^* catalytic mutant mice, increased inflammatory and immune signalling pathways were not among the top significantly enriched pathways. This may be due to elevated inflammatory and IFN-I signalling in tumour bearing *Parp7^HA/HA^* mice that is not further increased at injection of *Parp7* deficient cancer cells. This is supported by the gene expression profiling of CR705^Cas9^ tumours in *Parp7^HA/HA^* and C57BL/6 mice which revealed increased immune and IFN-I signalling in tumours from *Parp7^HA/HA^* mice. The increased inflammation of tumour bearing *Parp7^HA/HA^* mice, most likely contributes to the reduced growth of Cas9 and *Parp7* deficient tumours in *Parp7^HA/HA^* compared with C57BL/6 mice.

For tumour studies in C57BL/6 mice the observed increase in inflammatory gene profile most likely contributes to the increased tumour infiltration of T cells and NK cells. Within the T cell compartment, we observed increased levels of CD8^+^ T cells and CD4^+^ T_EM_ cells, but lower levels of Tregs in CR705^Parp7KO^ tumours. Reduced levels of anti-inflammatory M2 macrophages were also observed in CR705^Parp7KO^ compared with CR705^Cas9^ tumours. We also detected a lower relative abundance of MDSC populations and CD4^+^ Tregs within CR705^Parp7KO^ tumours, further suggesting a shift towards a less immunosuppressive TIL repertoire. While MDSC populations did express F4/80, they also exhibited high side scatter and were positive for Siglec-F. These results motivated us to classify these cells as granulocytes rather than traditional macrophages. F4/80 is commonly used to classify macrophages, but because of its expression on multiple immune cells, we propose that using F4/80 as a marker of only macrophages might overestimate the levels of these cells in IHC samples and flow cytometry samples without further analysis. We also observed increased populations of eosinophils in CR705^Parp7KO^ tumours. However, the exact roles of granulocyte populations in cancer are complex, as they can exhibit both pro- and anti-tumorigenic functions that vary by cancer type (61, 62). Additional studies are needed to determine the importance of eosinophils and other granulocytes in PARP7i-dependent antitumour immunity as well as in PDAC. Collectively, the increased infiltration of these immune cells most likely contributes to the increased anti-tumour immunity and reduced tumour progression of CR705^Parp7KO^ tumours.

Since subcutaneous injection of cancer cells, as was done in this study, does not accurately recapitulate the complex interactions and cell composition of TME that occur in orthotopic injection models or in KPC mouse models, the observed TIL profile is certainly influenced by the method used. It will be necessary to comprehensively investigate the role of PARP7 and its inhibition in more complex PDAC models that better reflect human disease. Collectively, our findings suggest that *Parp7* deficiency in cancer cells and or in other cells within the TME increases infiltration of immune cells and enhances anti-tumour activity. The data also suggest that PARP7 may be a suitable target for pancreatic cancer therapy, either alone or in combination with traditional immunotherapy strategies.

## Data Availability

The datasets presented in this study can be found in online repositories. The names of the repository/repositories and accession number(s) can be found below: https://www.ncbi.nlm.nih.gov/geo/query/acc.cgi?acc=GSE276293, GSE276293 and https://www.ncbi.nlm.nih.gov/geo/query/acc.cgi?acc=GSE284188, GSE284188.
