# Supplementary material for: Loss of Parp7 increases type I interferon signalling and reduces pancreatic tumour growth by enhancing immune cell infiltration
Source: Front Immunol. 2025 Jan 10;15:1513595. doi: 10.3389/fimmu.2024.1513595 (PMC11759301; doi:10.3389/fimmu.2024.1513595)
Supplement: Supplementary file 1 [file Image1.pdf]

WT Parp7

GAATTCGGCTTTGCAGATTTTTGCATAGCTTTTGAATCTTCATTTCTCAGTTTAAAAAAAGAAAATTGACCTGTA
AGAGCTAACTATAATGCAAGCAGTGATTGCAGATAGTTAACCATAGACTAACGCAAAATGTTTTAATGAATGAAT
GGGTTCAGTTGTCAGTTTTTAAATGATCATCTTTCTTCCTTTCCTGGTAGGATTTGTAGATACTGAGGCACAGTT
GGGAGTTAATCACATC**ATG**GAAGTGGAAACCACTGAACCTGAGCCAGACTGTGTAGTACAGCCTCCTTCTCCTTC
TGATGACTTTTCATGCCA**AATGAGAATTTCTGAGAAGA**TCTCTCCATTGAAAACGTGTTTTAAGAAAAAACAGGA
ACAAAAAAGATTGGGAACTGGAACCCTGAGATCCTTGAGGCCAATATTAAATACTTTGCTAGAATCTGGCTCACT
TGATGGAGTTTTTAGAGCTAGAGACCAAAACAGAGATGAGAGCAGCTTACATGAACATATAGTGAAAAAACCCCT
GGAAATCAACCCATCGTGTCCACCAGCAGAAAACAGTATGCCTGTCCTGATTCCTGATGGGACAAATGTTGAGGG
CCAATTACCAGAAGCGCATCCTTCTACAGATGCTCCAGAACAGGGGGTTCCAATCCAAGACCACAGTTTTCCACC
AGAAACCATCAGTGGGACAGTGGCAGATTCTACAACAGGACACTTCCAAACTGACCTTTTGCATCCTGTTTCAGG
TGATGTTCCTACAAGTCCTGACTGCGTAGATAAAGTTATGGATTATGTACCAGGAGCTTTCCAAGACAAAAGCCG
AATTC

Missing 104 (7/17)

GAATTCGGCTTTGCAGATTTTTGCATAGCTTTTGAATCTTCATTTCTCAGTTTAAAAAAAGAAAATTGACCTGTA
AGAGCTAACTATAATGCAAGCAGTGATTGCAGATAGTTAACCATAGACTAACGCAAAATGTTTTAATGAATGAAT
GGGTTCAGTTGTCAGTTTTTAAATGATCATCTTTCTTCCTTTCCTGGTAGGATTTGTAGATACTGAGGCACAGTT
GGG**AGAAGA**TCTCTCCATTGAAAACGTGTTTTAAGAAAAAACAGGAACAAAAAAGATTGGGAACTGGAACCCTGA
GATCCTTGAGGCCAATATTAAATACTTTGCTAGAATCTGGCTCACTTGATGGAGTTTTTAGAGCTAGAGACCAAA
ACAGAGATGAGAGCAGCTTACATGAACATATAGTGAAAAAACCCCTGGAAATCAACCCATCGTGTCCACCAGCAG
AAAACAGTATGCCTGTCCTGATTCCTGATGGGACAAATGTTGAGGGCCAATTACCAGAAGCGCATCCTTCTACAG
ATGCTCCAGAACAGGGGGTTCCAATCCAAGACCACAGTTTTCCACCAGAAACCATCAGTGGGACAGTGGCAGATT
CTACAACAGGACACTTCCAAACTGACCTTTTGCATCCTGTTTCAGGTGATGTTCCTACAAGTCCTGACTGCGTAG
ATAAAGTTATGGATTATGTACCAGGAGCTTTCCAAGACAAAAGCCGAATTC

Missing 31 (10 of 17)

GAATTCGGCTTTGCAGATTTTTGCATAGCTTTTGAATCTTCATTTCTCAGTTTAAAAAAAGAAAATTGACCTGTA
AGAGCTAACTATAATGCAAGCAGTGATTGCAGATAGTTAACCATAGACTAACGCAAAATGTTTTAATGAATGAAT
GGGTTCAGTTGTCAGTTTTTAAATGATCATCTTTCTTCCTTTCCTGGTAGGATTTGTAGATACTGAGGCACAGTT
GGGAGTTAATCACATC**ATG**GAAGTGGAAACCACTGAACCTGAGCCAGACTGTGTAGTACAGCCTCCTTCTCCTTC
TGATGACTCTCTCCATTGAAAACGTGTTTTAAGAAAAAACAGGAACAAAAAAGATTGGGAACTGGAACCCTGAGA
TCCTTGAGGCCAATATTAAATACTTTGCTAGAATCTGGCTCACTTGATGGAGTTTTTAGAGCTAGAGACCAAAAC
AGAGATGAGAGCAGCTTACATGAACATATAGTGAAAAAACCCCTGGAAATCAACCCATCGTGTCCACCAGCAGAA
AACAGTATGCCTGTCCTGATTCCTGATGGGACAAATGTTGAGGGCCAATTACCAGAAGCGCATCCTTCTACAGAT
GCTCCAGAACAGGGGGTTCCAATCCAAGACCACAGTTTTCCACCAGAAACCATCAGTGGGACAGTGGCAGATTCT
ACAACAGGACACTTCCAAACTGACCTTTTGCATCCTGTTTCAGGTGATGTTCCTACAAGTCCTGACTGCGTAGAT
AAAGTTATGGATTATGTACCAGGAGCTTTCCAAGACAAAAGCCGAATTC

Supplementary Figure S1. Indels resulting in reading frame errors in the *Parp7* gene. Genomic DNA was isolated from multiple selected and expanded clones, and the region surrounding the gRNA target site in *Parp7* (bold red font) was amplified and sequenced. The sequences above show the deletions (and frequencies) from the CR705^{Parp7KO} clone used in the study. The start ATG is in bold font. The two nucleotides flanking the deleting sequences are underlined.
