# Supplementary material for: Loss of Parp7 increases type I interferon signalling and reduces pancreatic tumour growth by enhancing immune cell infiltration
Source: Front Immunol. 2025 Jan 10;15:1513595. doi: 10.3389/fimmu.2024.1513595 (PMC11759301; doi:10.3389/fimmu.2024.1513595)
Supplement: Supplementary file 2 [file Image2.pdf]

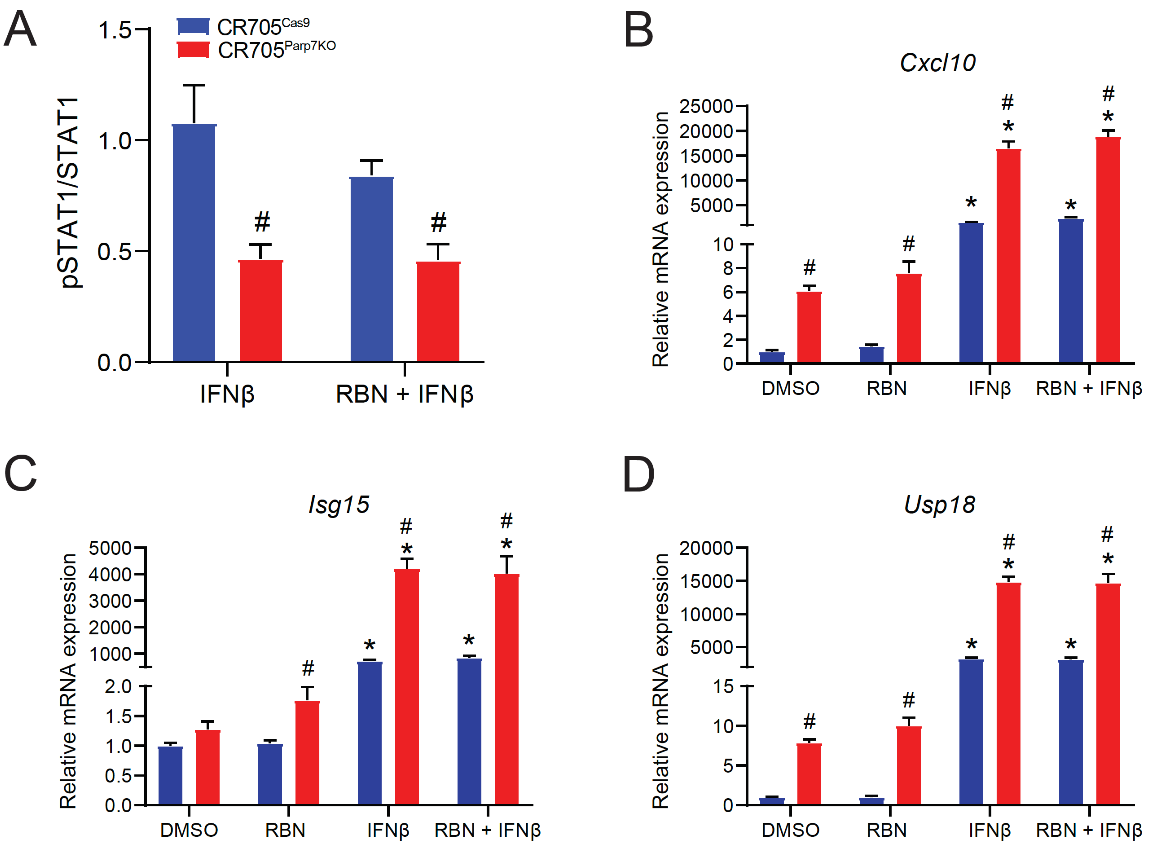

Supplementary Figure S2. Responses to exogenous IFNβ. **(A)** Relative pSTAT1 levels. Quantification of pSTAT1 bands relative to native STAT1 bands (pSTAT1/STAT1). Cells were pre-treated with 100 nM of RBN-2397 for 24 h and exposed to 1000 U/mL of IFNβ for 1 h. **(B-D)** Split axis showing increased basal expression levels of *Cxcl10*, *Isg15* and *Usp18* in Parp7^{KO} cells. Cells were pre-treated with RBN-2397 for 24 h and exposed to 1000 U/mL of IFNβ for 4 h. *$p<0.05$ denotes statistical significance compared with DMSO treated samples, #$p<0.05$ denotes significance due to loss of PARP7.
