# Supplementary material for: Loss of Parp7 increases type I interferon signalling and reduces pancreatic tumour growth by enhancing immune cell infiltration
Source: Front Immunol. 2025 Jan 10;15:1513595. doi: 10.3389/fimmu.2024.1513595 (PMC11759301; doi:10.3389/fimmu.2024.1513595)
Supplement: Supplementary file 3 [file Image3.pdf]

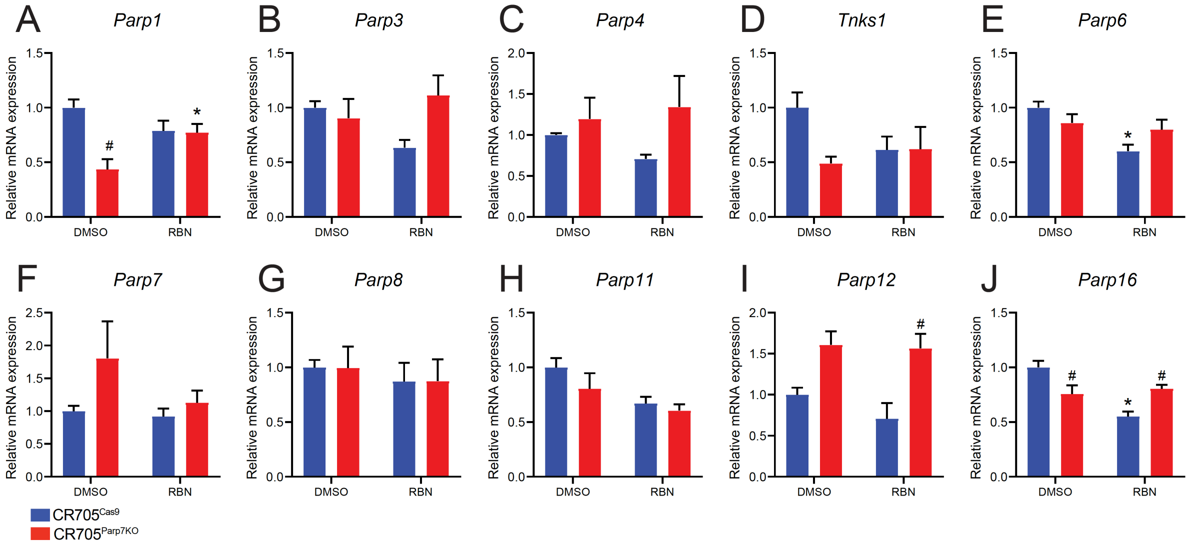

Supplementary Figure S3. Expression levels of ARTD family members in CR705^{Cas9} and CR705^{Parp7KO} cells after treatment with RBN-2397. (A) Levels of *Parp1* were decreased in the DMSO-treated knockout cells. (B-D, F-H) Levels of *Parp3*, *Parp4*, *Tnks1*, *Parp6*, *Parp7*, *Parp8* and *Parp11* were not significantly affected by loss or inhibition of PARP7. (E) *Parp6* expression was lower in the CR705^{Cas9} cells treated with RBN-2397. (J) *Parp12* levels were significantly elevated in the CR705^{Parp7KO} cells after treatment with RBN-2397. (J) Levels of *Parp16* were significantly lower in the DMSO treated CR705^{Parp7KO} cells, and in CR705^{Cas9} cells treated with RBN-2397. Cells were treated with DMSO or 100 nM of RBN-2397 for 24 h, and expression levels were determined with RT-qPCR. *$p<0.05$ denotes statistical significance from the DMSO treated samples, #$p<0.05$ significance due to loss of *Parp7*.
