# Supplementary material for: Loss of Parp7 increases type I interferon signalling and reduces pancreatic tumour growth by enhancing immune cell infiltration
Source: Front Immunol. 2025 Jan 10;15:1513595. doi: 10.3389/fimmu.2024.1513595 (PMC11759301; doi:10.3389/fimmu.2024.1513595)
Supplement: Supplementary file 4 [file Image4.pdf]

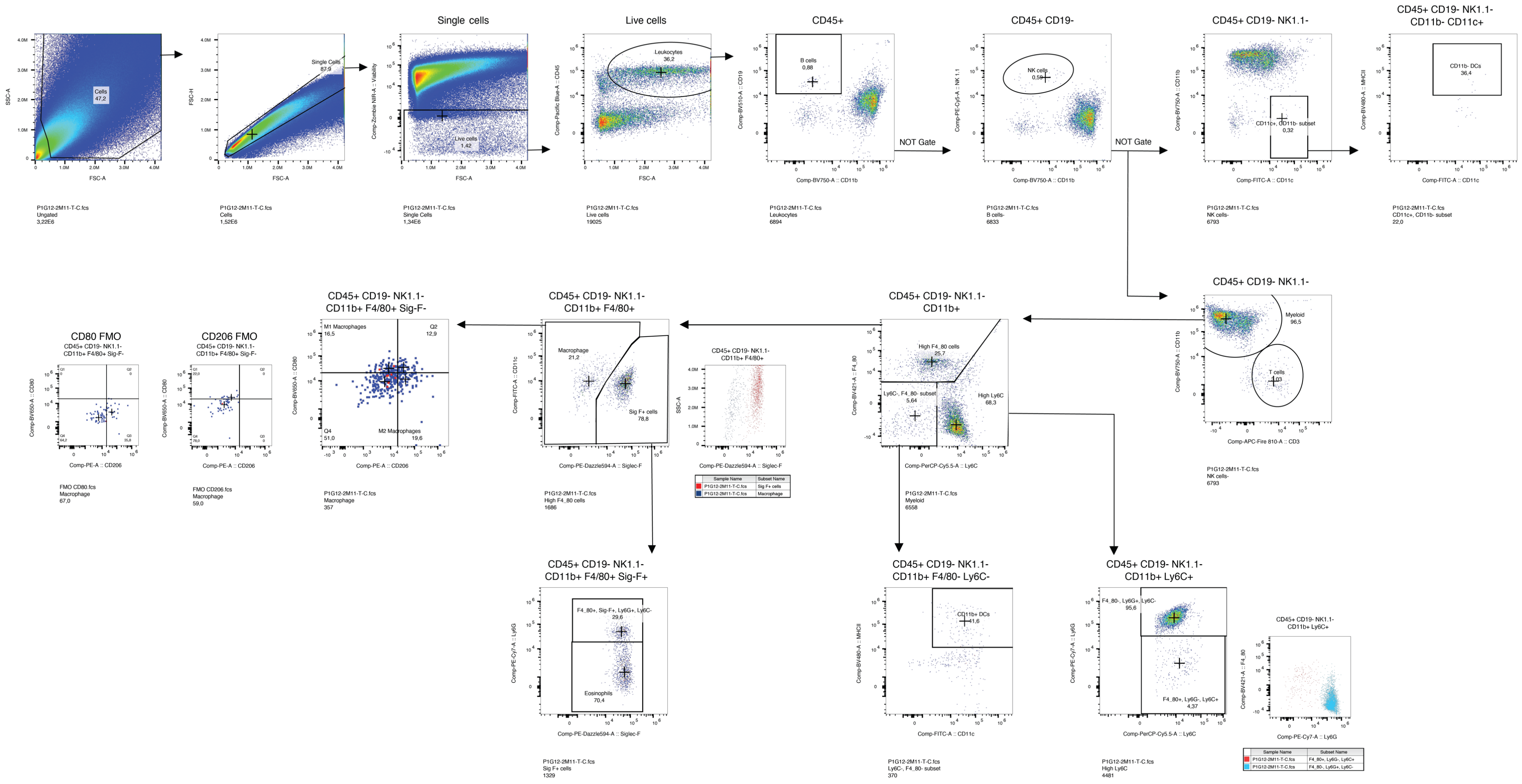


**Supplementary Figure S4.** Gating strategy for flow cytometry analysis of leukocytes from single cell suspensions of CR705$^{Cas9}$ and CR705$^{Parp7KO}$ tumours. NOT gates include all cells that are outside the gate shown.
