# Supplementary material for: Loss of Parp7 increases type I interferon signalling and reduces pancreatic tumour growth by enhancing immune cell infiltration
Source: Front Immunol. 2025 Jan 10;15:1513595. doi: 10.3389/fimmu.2024.1513595 (PMC11759301; doi:10.3389/fimmu.2024.1513595)
Supplement: Supplementary file 5 [file Image5.pdf]

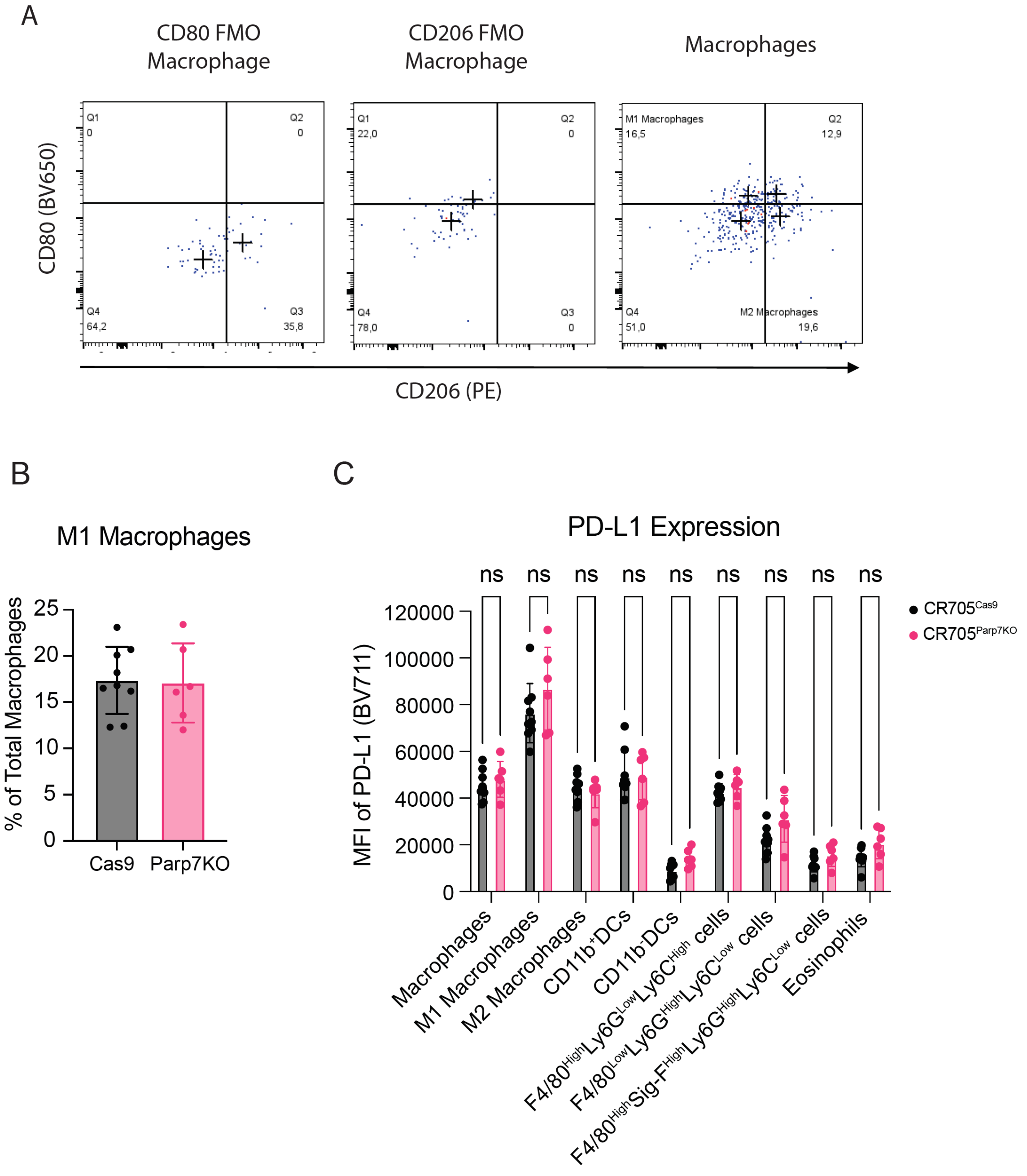

Supplementary Figure S5. Flow cytometry analysis of macrophage phenotypes in CR705^{Cas9} and CR705^{Parp7KO} tumours. **(A)** Analysis of M1-like (CD80^{hi}, CD206^{low}) pro-inflammatory or M2-like (CD80^{low}, CD206^{hi}) anti-inflammatory phenotype and macrophages in tumours. **(B)** M1 macrophages from CR705^{Cas9} and CR705^{Parp7KO} tumours. **(C)** No differences in the expression of the immune checkpoint PD-L1 were observed across all tumour infiltrating leukocyte (TIL) populations between CR705^{Cas9} and CR705^{Parp7KO} tumours.
