# Supplementary material for: Loss of Parp7 increases type I interferon signalling and reduces pancreatic tumour growth by enhancing immune cell infiltration
Source: Front Immunol. 2025 Jan 10;15:1513595. doi: 10.3389/fimmu.2024.1513595 (PMC11759301; doi:10.3389/fimmu.2024.1513595)
Supplement: Supplementary file 6 [file Image6.pdf]

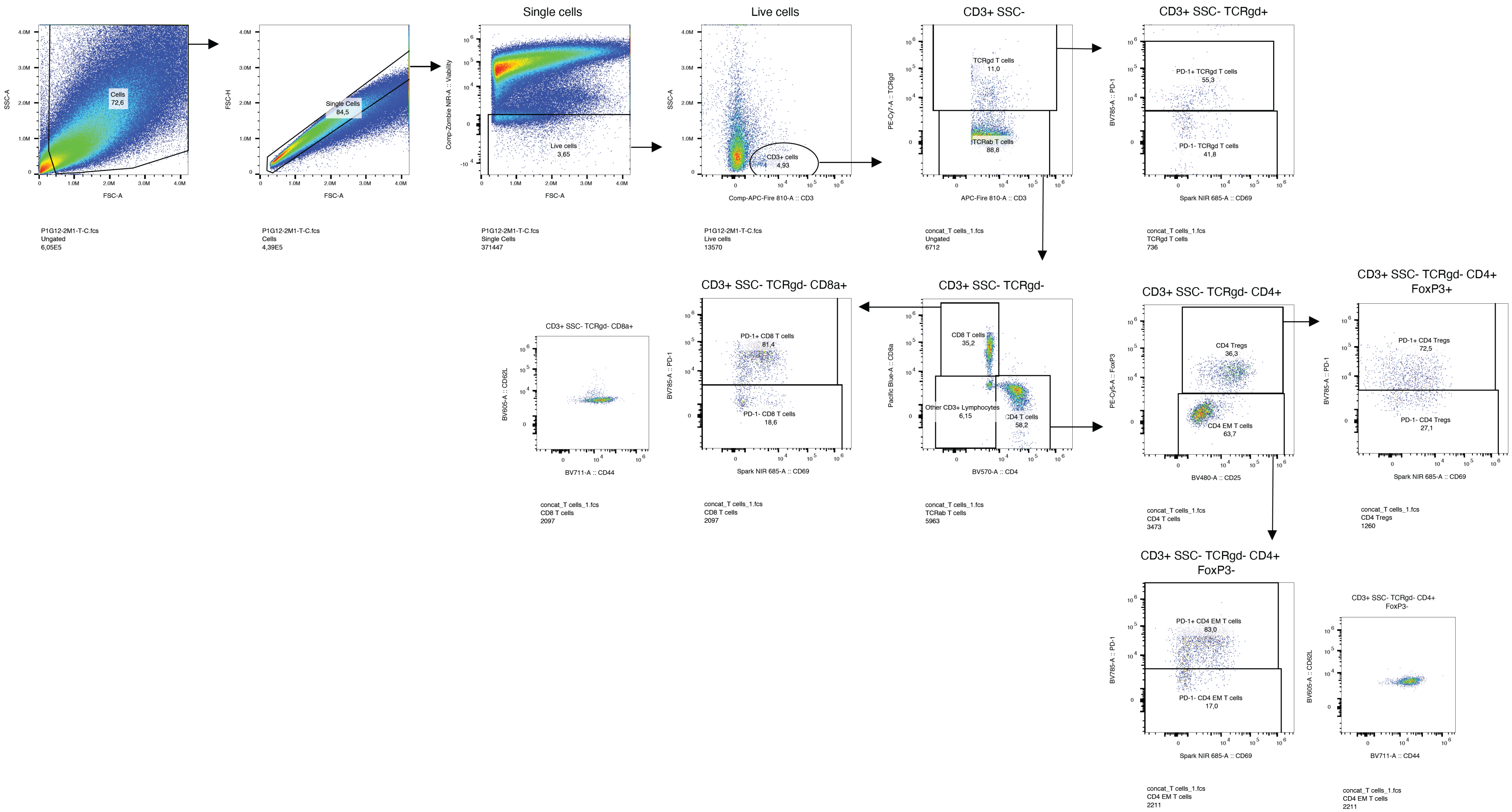

Supplementary Figure S6. Gating strategy for flow cytometry analysis of T cell populations from single cell suspensions of CR705^{Cas9} and CR705^{Parp7KO} tumours.
