# Supplementary material for: Loss of Parp7 increases type I interferon signalling and reduces pancreatic tumour growth by enhancing immune cell infiltration
Source: Front Immunol. 2025 Jan 10;15:1513595. doi: 10.3389/fimmu.2024.1513595 (PMC11759301; doi:10.3389/fimmu.2024.1513595)
Supplement: Supplementary file 7 [file Table1.docx]

**Supplementary Table S1.** Primers used for RT-qPCR.

| *Gene* | Strand | Sequence |
| --- | --- | --- |
| *Tbp* | Forward  Reverse | GCACAGGAGCCAAGAGTGAA  TAGCTGGGAAGCCCAACTTC |
| *Ifnb1* | Forward  Reverse | TGGGAGATGTCCTCAACTGC  CCAGGAGTAGCTGTTGTACT |
| *Cyp1a1* | Forward  Reverse | CGTTATGACCATGATGACCAAGA  TCCCCAAACTCATTGCTCAGAT |
| *Cxcl10* | Forward  Reverse | CCAAGTGCTGCCGTCATTTTC  GGCTCGCAGGGATGATTTCAA |
| *Stat1* | Forward  Reverse | GCCTCTCATTGTCACCGAAGAAC  TGGCTGACGTTGGAGATCACCA |
| *Stat2* | Forward  Reverse | GAACCAACTCTCCATTGCCTGG  CGTAAGAGGAGAACTGCCAGCT |
| *Irf9* | Forward  Reverse | CAACATAGGCGGTGGTGGCAAT  GTTGATGCTCCAGGAACACTGG |
| *Isg15* | Forward  Reverse | CATCCTGGTGAGGAACGAAAGG  CTCAGCCAGAACTGGTCTTCGT |
| *Usp18* | Forward  Reverse | GGAACCTGACTAAGGACCAGATC  GAGAGTGTGAGCAGTTTGCTCC |
| *Parp1* | Forward  Reverse | GGCAGCCTGATGTTGAGGT  GCGTACTCCGCTAAAAAGTCAC |
| *Parp2* | Forward  Reverse | TGGAAGGCGAGTGCTAAATG  GGGCTTTGCCCTTTAACAGC |
| *Parp3* | Forward  Reverse | TGCGGCATGTTTGGAAAGTG  GTGCATGGTGGTAACATAGCC |
| *Parp4* | Forward  Reverse | AGTGCTACAGCCCGTTTCC  CACAGCTTTCAGTTGTGGGC |
| *Tnks1* | Forward  Reverse | CCCTGAGGCCTTACCTACCT  TCAAGACCCGCAACTTCTCC |
| *Tnks2* | Forward  Reverse | TGATGGCAGAAAGTCAACTCCA  GCCACAGGTCCATTGCATTC |
| *Parp6* | Forward  Reverse | GTACCTTGATGGACCAGAGCC  GCCAGCTCGGAACTTCTTGA |
| *Parp7* | Forward  Reverse | AAAACCCCTGGAAATCAACC  GAATCTGCCACTGTCCCACT |
| *Parp8* | Forward  Reverse | CACTTCCGAAACCACTTCGC  TAGGATACACTTTTGGGGCCG |
| *Parp9* | Forward  Reverse | GCATTTGCTAAAGAGCACAAGGA  AAAGCACCACTATTACCGCTGA |
| *Parp10* | Forward  Reverse | CGAAACGGCACACTCTACGG  GAGACCCTCAAAGGAGGTGC |
| *Parp11* | Forward  Reverse | CAAACCCTTGTGGCTCCATTTCC  AGGCACTGATGGAGAAAGGAGC |
| *Parp12* | Forward  Reverse | AAGTTCTGACCTGGTGAGCAGG  TGGTGACACAGGACCTGAACTG |
| *Parp13* | Forward  Reverse | AGTAGTCCCACTGGTTTTGGC  TGCAACTCTGTGGCTTGTGG |
| *Parp14* | Forward  Reverse | TGCTGAAGCTGTCAAGACTACA  ACAATGGCATGGGTCGTAGC |
| *IFNB* | Forward  Reverse | ATGACCAACAAGTGTCTCCTCC  GCTCATGGAAAGAGCTGTAGTG |
| *CYP1A1* | Forward  Reverse | TGGTCTCCCTTCTCTACACTCTTGT  ATTTTCCCTATTACATTAAATCAATGGTTCT |
| *TBP* | Forward  Reverse | TTGTACCGCAGCTGCAAAAT  TATATTCGGCGTTTCGGGCA |
